# The Effect of Contrast Reversal on Peripheral Visual Acuity

**DOI:** 10.1167/tvst.14.8.23

**Published:** 2025-08-19

**Authors:** Carlos R. Cassanello, Neil W. Roach, Chris Scholes, Paul V. McGraw

**Affiliations:** 1 School of Psychology, University of Nottingham, Nottingham, UK

**Keywords:** peripheral vision, contrast reversal, visual acuity, central vision loss, macular degeneration

## Abstract

**Purpose:**

Patients with central vision loss must rely upon regions of their unaffected peripheral retina to carry out fine-scale visual tasks. We aimed to determine the conditions under which contrast reversal of targets and/or backgrounds may act to improve visual acuity in the peripheral field.

**Methods:**

Ten participants with normal or corrected-to-normal vision performed a visual acuity task in which they were asked to identify the orientation of a letter-C target presented at 10° in the right visual field. Minimum letter size thresholds were measured for static (black or white) and contrast-reversing (8.5 or 17 Hz) targets presented on a uniform mean luminance background, a static patterned background, or a dynamic version of the patterned background, which was contrast reversing at the same temporal frequencies used for the targets.

**Results:**

When presented on a uniform background, black targets produced lower thresholds than white targets, and there was no consistent benefit of contrast reversal. However, when presented on a patterned background, acuity for contrast-reversing targets consistently exceeded that for either polarity static target. Contrast reversing the background also improved acuity, for both static targets and targets that contrast reversed at a different rate.

**Conclusions:**

Peripheral visual acuity is significantly enhanced by dynamic contrast modulation, particularly when the modulation acts to promote segmentation between target and background.

**Translational Relevance:**

Adding temporal modulation to the target or background could provide a simple way of enhancing peripheral acuity for patients with central vision loss.

## Introduction

Many visual tasks, such as identifying objects, reading text, and recognizing faces, involve the identification and fine discrimination of visual features. This spatial capacity is typically quantified using a stress test, where the dimensions of a target are reduced until recognition or discrimination performance fails. The most common measure used in clinical settings to quantify spatial performance is visual acuity, measured using optotypes as targets. Visual acuity in the normal human retina peaks in the central macular region, where the density of retinal cone photoreceptors is highest and where convergence onto ganglion cells is lowest.^1^^–^^5^ Beyond the macular region, the ability to discriminate fine spatial details progressively (and rapidly) declines.^4^^,^^6^^–^^8^

Patients who develop central vision loss, such as those affected by age-related macular degeneration (ARMD), have structural damage to the macular region that results in severely reduced visual acuity and a loss of visual sensitivity. This is usually caused by the continual aggregation of protein deposits called drusen (dry-ARMD) or the proliferation (and leakage) of abnormal new blood vessels into the macular region (wet-ARMD). Consequently, in both wet and dry forms of ARMD, patients’ ability to perform visual tasks that require good visual acuity is significantly impaired.

When macular function is lost, individuals often adopt a more peripheral retinal region in order to view objects—termed a preferred retinal locus (PRL). It is still unclear what governs the choice of a PRL, and patients with central vision loss can develop distinct PRLs to support different visual tasks.^9^^–^^14^ However, the PRL is located at a greater eccentricity than the fovea and thus suffers from the known limitations to spatial vision in the peripheral field, such as a lower density of cones, increased pooling of signals onto ganglion cells, and a smaller cortical representation, producing functional bottlenecks such as increased visual crowding and reduced visual acuity.^4^ Therefore, when central visual function is lost, finding ways to maximize peripheral acuity becomes a priority.

Previous work from our group demonstrated that adding temporal modulations to a target improved dynamic visual acuity measured at 10° in the horizontal peripheral field.^15^^,^^16^ For a smoothly moving peripheral target translating along an isoeccentric arc, both temporally subsampling the image and periodically reversing its contrast polarity were effective in lowering thresholds at higher target speeds (10–20°/s). However, for static or slowly moving targets, image subsampling impaired performance, whereas contrast polarity reversal continued to be beneficial. A consistent improvement in acuity threshold was found for static and dynamic contrast-reversing targets presented on a gray background versus equivalent white targets presented on the same background.

In the present study, we now focus on the effects of contrast polarity for static acuity targets and extend the previous work in a number of ways. First, we test a broader range of target configurations for both static and contrast-reversing targets and examine whether the relative rate of contrast reversal is important. We also used two types of backgrounds, a uniform space-averaged luminance field and a patterned background containing noise with an inverse square law spatial frequency profile. Finally, we investigated the effect of contrast reversing the patterned background, either at the same rate as the target or at a different rate.

## Methods

### Participants

Ten participants were recruited from students and faculty of the School of Psychology, including the authors and six individuals who were naive to the specific purposes of the study (eight men, two women, age range 22–64). All participants gave informed consent before participation, and the experimental procedures were consistent with the principles of the Declaration of Helsinki. The study was approved by the Ethics Committee of the School of Psychology at the University of Nottingham.

### Stimuli and Procedure

The experiment and stimuli were controlled and presented using Python (Psychopy) and presented on a gamma-corrected NEC Multisync CRT FP2141-SB monitor (resolution = 1280 × 1024 pixels; frame rate = 85 Hz; mean background luminance = 45 cd/m^2^; visual angle = 0.98′/pixel).

**Figure 1.**
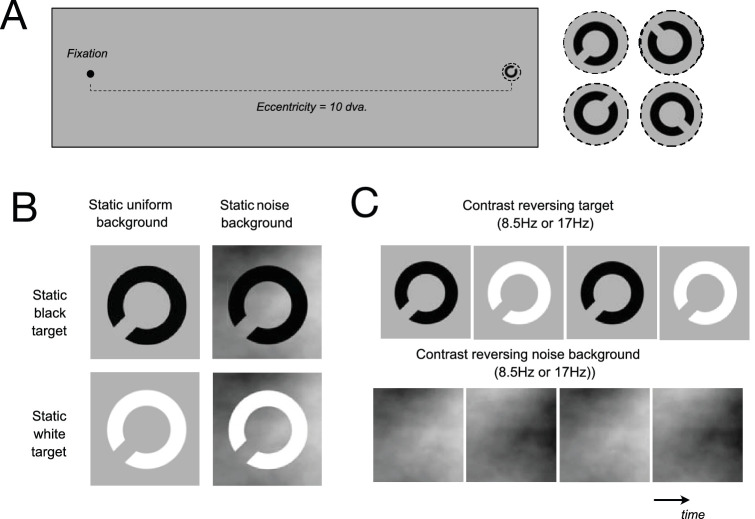
Schematic of the task and stimuli. (**A**) The fixation point was located on the vertical midline of the screen and at 8° to the left of the center of the screen. Letter C targets were presented at 10° eccentricity to the right of the fixation point. The background (not shown) was centered at the target location so that the target was always in the middle of the background. On a given trial, participants were asked to identify the orientation of the gap in the letter C from one of four alternatives. (**B**) In static conditions, the target remained either black or white throughout the trial and was presented on either a uniform gray background or on a background comprising noise randomly generated on each trial. (**C**) In dynamic conditions, the target and/or the background reversed contrast polarity at one of two rates.

Participants were seated in a darkened room at a viewing distance of 114 cm from the monitor, with their heads supported by a chinrest. Viewing was binocular, and participants were asked to hold fixation on a black cross. As shown in Figure 1A, a letter C (Sloan font) was presented on each trial for 530 ms at 10° eccentricity in the right visual field. The target was presented in one of four orientations (±45°, ±135°) and participants indicated the orientation of the gap of the C by pressing one of four keys, 1, 4, 5, or 2, for lower-left, upper-left, upper-right, or lower-right, respectively. Following each response, auditory feedback was provided (correct responses = high-pitched tone, incorrect responses = low-pitched tone), and the next trial was initiated. The size of the target varied according to a 3-down, 1-up staircase starting from 1.5° diameter for the whole optotype, changing step sizes with each reversal, following the sequence 0.4°, 0.2°, 0.2°, 0.1°, 0.1°, 0.05°, 0.05°, 0.025°, 0.025°.

Each staircase consisted of a minimum of 50 trials and nine reversals, and a minimum of six staircases were completed by each participant for each condition. Thresholds for each staircase were estimated by averaging across the final six reversals.

The experiment consisted of a total of 16 conditions where both target and background appearance were manipulated (see Fig. 1B). The target could be black (B), white (W), or undergo square wave contrast polarity reversal (CR) at either 17 Hz (H) or 8.5 Hz (L). In the reversing conditions, the initial contrast polarity was randomly selected as either B or W. The background could be uniform gray (U) or contain a structured noise texture (N). Noise textures were composed of grayscale elements with a base spatial frequency of 4 cycles/degree and a 1-octave bandwidth, filtered to produce a power-law spectrum with a 1/f² profile (Brown or Brownian noise) with a lower bound of 0 cycles/degree and upper bound of 6 cycles/degree. Brownian noise was chosen to minimize the spectral overlap with the critical features of the letter C target. The noise-patterned backgrounds spanned an area of 16.7° by 16.7°, were centered on the target location, and had contrasts set to 90% of maximum. In different conditions, the noise background could remain static or reverse contrast at 17 Hz or 8.5 Hz. Separate staircases for each condition were run consecutively, but the ordering of conditions was randomly shuffled for each participant.

The 16 conditions can be split into four subgroups based on the dynamic states of targets and backgrounds. (1) Static targets and backgrounds (static on static): targets remained of fixed polarity throughout the trial (B or W), and backgrounds were also static, either uniform or noise (U or N). (2) Targets reversed polarity on static backgrounds (reversing on static): targets reversed polarity at 8.5 Hz (L) or 17 Hz (H) on backgrounds held static throughout each trial (U or N). (3) Static targets on reversing backgrounds (static on reversing): target polarity remained fixed (B or W) on backgrounds reversing contrasts at 8.5 Hz (L) or 17 Hz (H); note that in this case, only noise backgrounds (N) can be used. (4) Both targets and backgrounds reversed contrast at 8.5 Hz and 17 Hz (reversing on reversing): this contrast reversal could be synchronous with target and contrast-reversing contrast at the same frequency (LL or HH), or asynchronous, where the target reversed at a slower (LH) or faster (HL) rate than the background. We adopt a notation where the first letter indicates the dynamic state of the target and the second that of the background.

### Statistical Analysis

While all participants ran at least six staircases for each condition, two participants had more than six staircases in several conditions. As this resulted in unbalanced observation sets for each participant, we chose to use linear models for our analysis. Statistical analyses were conducted in R, using the packages lme4^17^ for linear mixed-model fitting, and easystats and emmeans^18^ to obtain model parameters, evaluate model performance, and compute post hoc contrasts. Linear mixed models were fitted to the participants with two four-level fixed-effect factors describing changes in target (B, W, L, and H) and background (U, N, L, and H) state. Participant ID was included as a random effect variable with staircase number nested within participant. Statistical significance was assessed through pairwise contrasts, corrected for multiple comparisons (Bonferroni method). The supplementary material contains an annotated version of the RStudio notebook that generates the analyses along with the data and model parameters.

## Results

**Figure 2.**
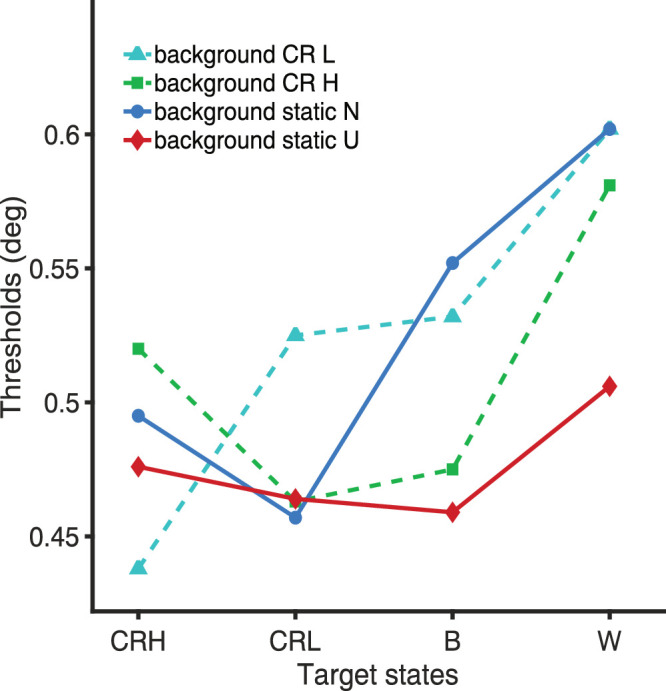
Group mean size thresholds for discriminating letter C targets under different target and background state conditions. Target states are shown on the x-axis, where CRH indicates target-reversing polarity at 17 Hz, CRL indicates target-reversing polarity at 8.5 Hz, B is target static black, and W is target static white. Background states are coded by the color, where *red* is a static uniform gray background, *blue* is a static noise background, *cyan-dashed* is a dynamic background contrast reversing at 8.5 Hz (CR L), and *green-dashed* is a dynamic background contrast reversing at 17 Hz (CR H). Thresholds ranged from worst to best condition from 0.602° to 0.438°, an improvement of 0.138 logMAR, or roughly seven letters on a logMAR chart.

Figure 2 shows how mean thresholds for the group varied across different combinations of target and background state. From worst to best condition, size thresholds ranged from 0.602° to 0.438°, an improvement of 0.138 logarithm of the minimum angle of resolution (logMAR), or roughly seven letters on a logMAR chart (each letter corresponds to 0.02 logMAR and each line on the chart to 0.1 logMAR). This change represents a strong and clinically relevant improvement in visual acuity. In general, differences in threshold below 0.025 logMAR (or one letter on the chart) resulted in statistical nonsignificance. While a full set of pairwise contrasts between conditions is available in the supplementary material, here we focus on how changes to the key subgroups of target and background state affect visual acuity.

### Better Acuity for Static Black Versus Static White Targets

**Figure 3.**
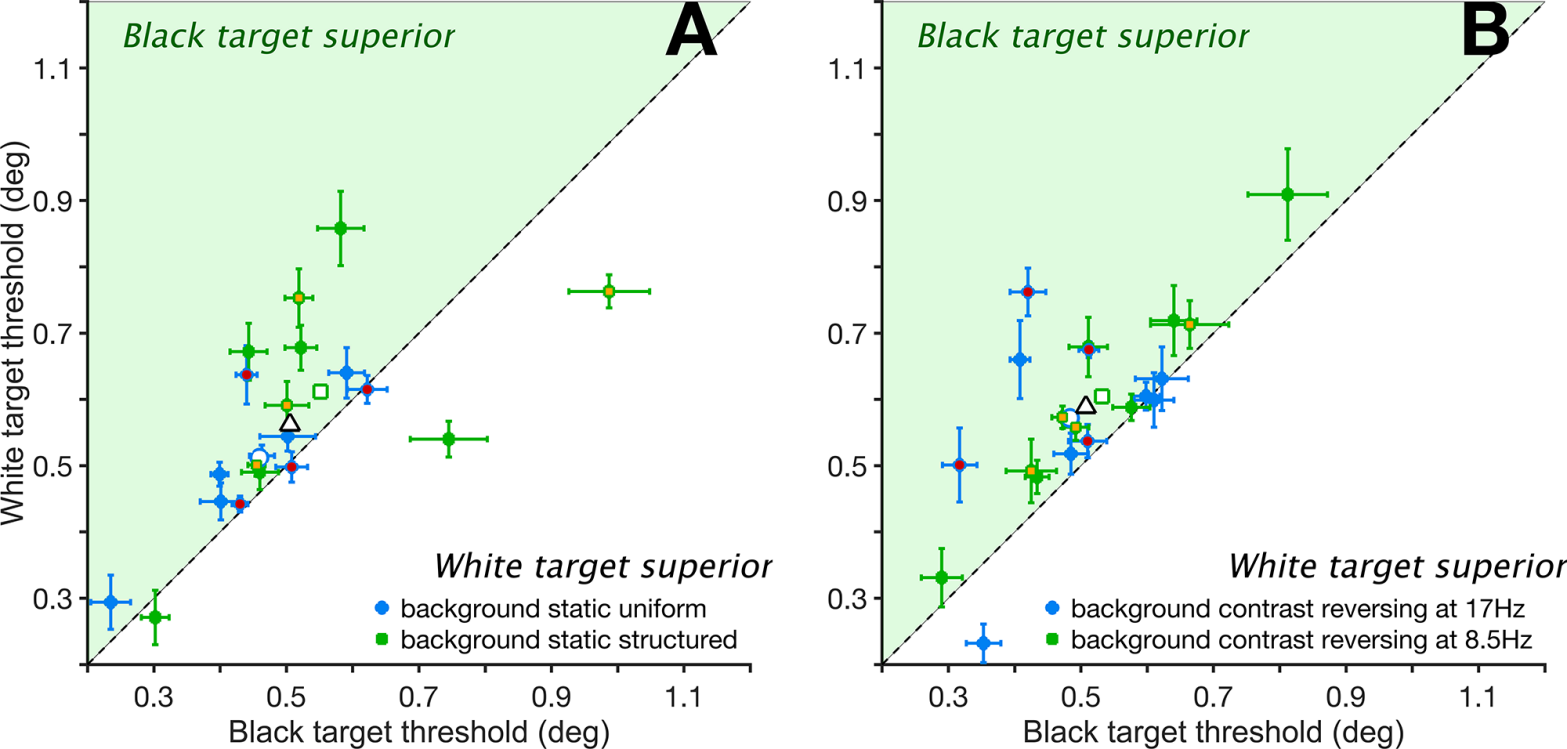
Size thresholds for white targets plotted as a function of those with black targets. *Filled points* indicate thresholds for individual participants with 95% confidence intervals. Datapoints falling above the diagonal indicate relatively better performance for black targets compared to white targets. (**A**) Data obtained with static backgrounds: *blue dots*, uniform background (U); *green dots*, static noise background (N). (**B**) Contrast-reversing backgrounds: *blue dots*, 17 Hz; *green dots*, 8.5 Hz. *Empty symbols* with similar edge color and shapes are means of their matching subgroups. The overall mean is the *empty triangle* with a black edge. Data from Supplementary Table S1. Data collected from the authors are symbols with different face color.

When the target remained unchanged throughout the trial (i.e., B or W), performance in conditions with B targets yielded lower thresholds than with W ones. This advantage was observed on both static and dynamic backgrounds, as shown in Figure 3.

In static conditions, B targets showed a mean threshold improvement of 0.049° over W targets on a uniform gray (U) background (0.459° vs. 0.506°; *P* < 0.001, *t* = 5.81; Fig. 3A, blue dots). On a structured noise (N) background, the mean B target advantage was 0.052° (0.552° vs. 0.602°; *P* < 0.001, *t* = 6.21; Fig. 3A, green dots).

In dynamic conditions where the N background reversed contrast (Fig. 3B), the performance advantage for B targets was more pronounced. At a 17-Hz contrast reversal, B targets outperformed W targets by 0.094° (0.475° vs. 0.581°; *P* < 0.001, *t* = 11.39; Fig. 3B, blue dots). At 8.5 Hz, the advantage was 0.072° (0.532° vs. 0.602°; *P* < 0.001, *t* = 8.62; Fig. 3B, green dots).

### Contrast Reversing the Target Produces No Consistent Benefit When Presented on a Static Uniform Background

**Figure 4.**
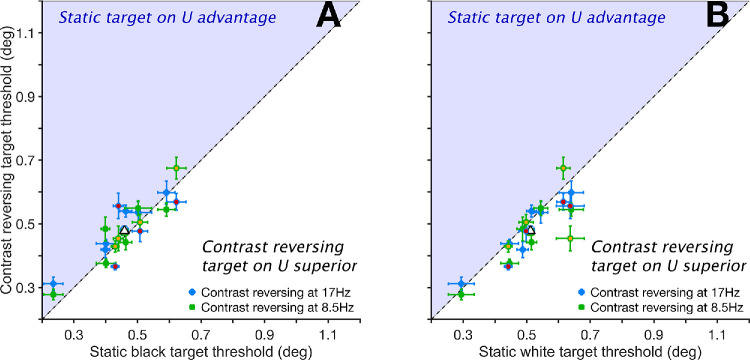
Size thresholds for contrast-reversing targets on a uniform (U) background plotted relative to those with static black (**A**) and white targets (**B**). *Error bars* are 95% confidence intervals. In both panels, *dots* above the diagonal (inside the *light blue shaded area*) indicate larger thresholds in the conditions where the target reversed contrast at 17 Hz (*blue dots*) or 8.5 Hz (*green dots*). *Empty symbols* with similar edge color and shapes are means of their matching subgroups. The overall mean is the *empty triangle* with a black edge. Data from Supplementary Table S1. Data collected from the authors are symbols with different face color.

Figure 4 shows thresholds where the target reversed contrast plotted against thresholds for the corresponding condition where the target remained static. Relative to static black (B) targets (Fig. 4A), mean thresholds for CR targets at 17 Hz (blue dots) were marginally higher (0.476° vs. 0.459°; *P* = 0.575, *t* = 2.82), as were those for CR targets at 8.5 Hz (green dots, 0.464 vs. 0.459°; *P* > 0.999, *t* = 1.90). When pooled, CR targets showed a nonsignificant difference of 0.020° compared to static B targets (0.470° vs. 0.459°; *P* = 0.112, *t* = 2.72).

Comparing CR targets to static white (W) targets (Fig. 4B), thresholds for CR targets were generally lower, indicating a small advantage. Pooled CR thresholds were 0.029° lower than static W targets (0.477° vs. 0.506°; *P* = 0.001, *t* = 3.99). This difference was significant at the lower reversal rate of 8.5 Hz (Fig. 4B, green dots), with CR targets showing 0.032° lower thresholds than W targets (0.474° vs. 0.506°; *P* = 0.010, *t* = 3.93). However, at 17 Hz (Fig. 4B, blue dots), the CR advantage of 0.025° (0.481 vs. 0.506°) was not statistically significant (*P* = 0.358, *t* = 2.97).

### Contrast Reversing the Target Improves Acuity When Presented on a Static Noise Background

**Figure 5.**
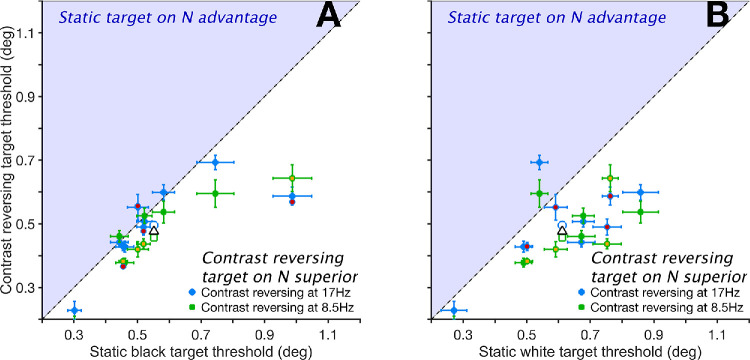
Size thresholds for contrast-reversing targets on a noise (N) background plotted relative to those with static black (**A**) and white targets (**B**). *Error bars* are 95% confidence intervals. In both panels, *dots* above the diagonal (inside the *light blue shaded area*) indicate larger thresholds in the conditions where the target reversed contrast at 17 Hz (*blue dots*) or 8.5 Hz (*green dots*). *Empty symbols* with similar edge color and shapes are means of their matching subgroups. The overall mean is the *empty triangle* with a black edge. Data from Supplementary Table S1. Data collected from the authors are symbols with different face color.

Figure 5 shows thresholds when targets were presented on a structured noise (N) background. CR targets showed clear performance advantages over static targets in this context. CR at 17 Hz yielded thresholds 0.056° lower than static black (B) targets (0.495° vs. 0.552°; *P* < 0.001, *t* = 6.59; Fig. 5A, blue dots). At 8.5 Hz, the advantage was larger at 0.095° (0.457° vs. 0.552°; *P* < 0.001, *t* = 11.14; Fig. 5A, green dots). CR targets also outperformed static white (W) targets (Fig. 5B) with a 0.108° threshold benefit at 17 Hz (0.495° vs. 0.602°; *P* < 0.001, *t* = 12.94) and a larger 0.147° threshold benefit at 8.5 Hz (0.457° vs. 0.602°; *P* < 0.001, *t* = 17.57).

Pooled across both reversal rates, CR targets outperformed B targets by 0.076° (0.476° vs. 0.552°; *P* < 0.001, *t* = 10.24) and W targets by 0.127° (0.476° vs. 0.602°; *P* < 0.001, *t* = 17.74). When all CR conditions were compared to all static target conditions on N backgrounds, thresholds for CR targets were 0.101° lower (0.476° vs. 0.577°; *P* < 0.001, *t* = 17.01). These findings highlight a marked benefit of contrast-reversing targets on structured backgrounds, in contrast to the minimal effect observed with uniform backgrounds (compare solid red and blue lines in Fig. 2).

### Fast Contrast Reversal of Noise Background Improves Acuity for Static Targets

**Figure 6.**
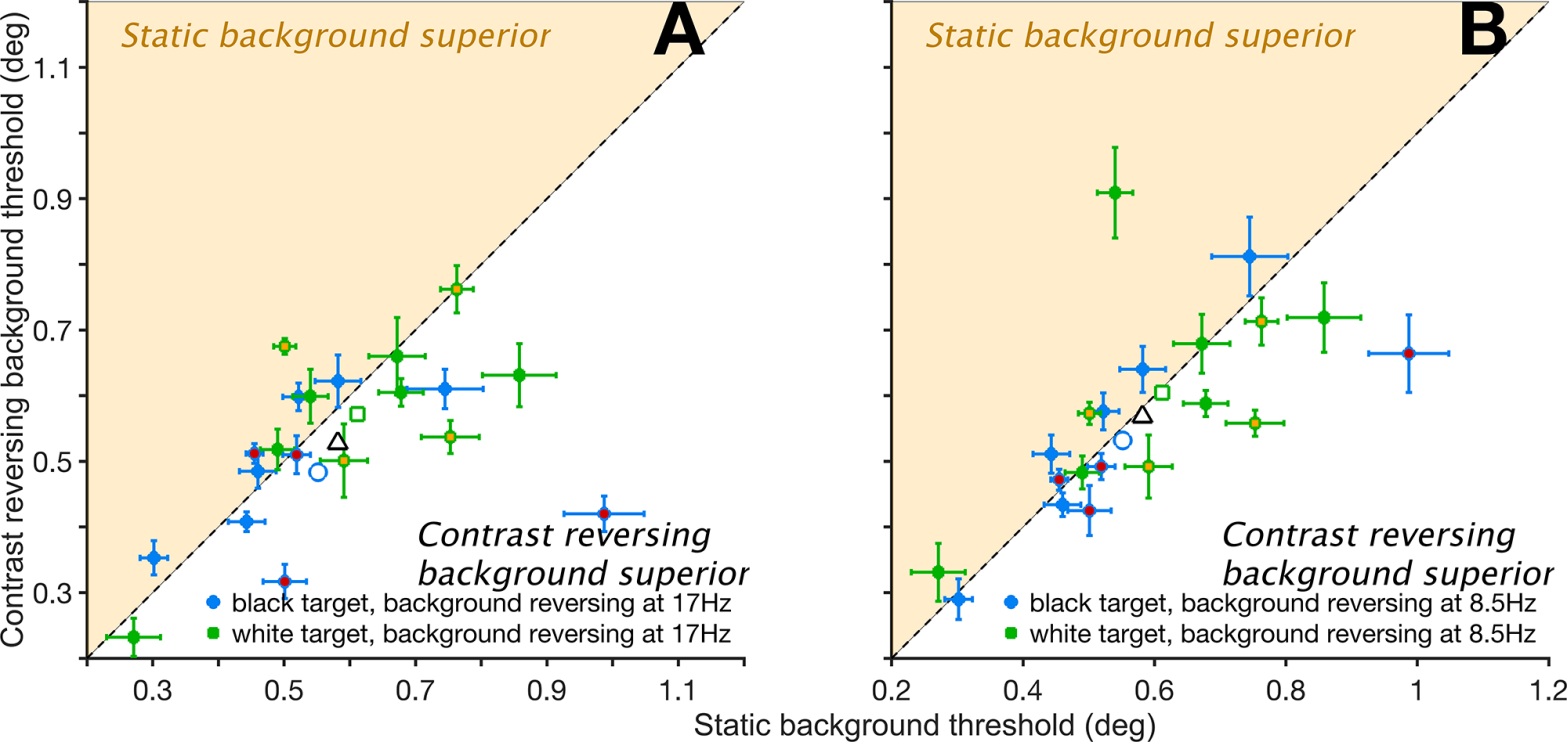
X-axis, both panels: Size thresholds obtained with black (*blue dots*, both panels) or white targets (*green dots*) presented on a static noise background (N). Y-axis: thresholds obtained when the background contrast reversed at either 17 Hz (**A**, y-axis) or 8.5 Hz (**B**). *Error bars* are 95% confidence intervals. In both panels, *dots* above the diagonal (inside the *light orange area*) indicate larger thresholds in the conditions where the background reversed contrast at 17 Hz (*left*) or 8.5 Hz (*right*). *Empty symbols* with similar edge color and shapes are means of their matching subgroups. The overall mean is the *empty triangle* with a black edge. Data from Supplementary Table S1. Data collected from the authors are symbols with different face color.

Figure 6 shows thresholds for static black (B) and white (W) targets presented on structured noise (N) backgrounds that either remained static or reversed contrast. When the background reversed contrast at 17 Hz (Fig. 6A), mean thresholds for B targets were 0.062° lower than in the static condition (0.475° vs. 0.552°; P < 0.001, *t* = 7.38), whereas for W targets, the difference was smaller (0.020°) and not significant (0.581° vs. 0.602°; *P* > 0.999, *t* = 2.48). When pooled across both target types, the benefit of background reversal at 17 Hz remained significant (*P* < 0.001, *t* = 7.03).

At the lower reversal rate of 8.5 Hz (Fig. 6B), the effect of background modulation was weaker and not statistically significant. For B targets, the threshold reduction was 0.020° (0.532° vs. 0.552°; *P* > 0.999, *t* = 2.35), while for W targets, there was virtually no change, with thresholds identical to the static condition (<0.001°; 0.602° vs. 0.602°; *P* > 0.999, *t* = 0.01). When data were pooled across target types at 8.5 Hz, the overall improvement in thresholds was 0.010° (0.567° vs. 0.577°; *P* > 0.999, *t* = 1.70), again not reaching significance.

### Asynchronous Contrast Reversal of Target and Background Improves Acuity

**Figure 7.**
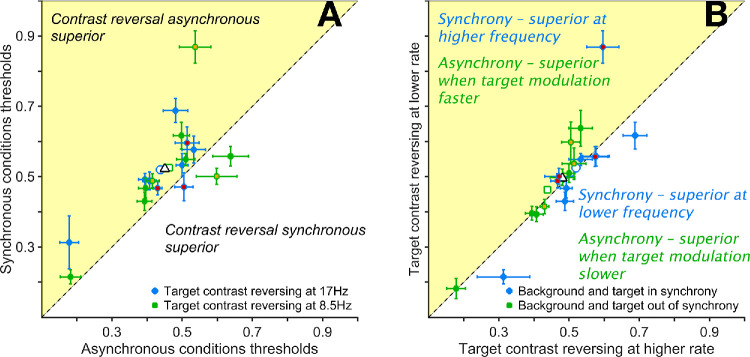
Size thresholds obtained with full dynamic conditions where targets and thresholds reversed contrast at the two rates tested, in and out of synchrony. The data shown in both panels are the same, but they have been arranged to plot thresholds of different conditions as a function of the other. (**A**) Synchronous versus asynchronous conditions. Size thresholds from synchronous conditions plotted as a function of those of the asynchronous condition, where the target reversed contrast at the same rate: *blue dots*, 8.5 Hz; *green dots*, 17 Hz. (**B**) Size thresholds of one condition as a function of the other within the synchronous group (*blue dots*) or asynchronous group (*green* *dots*). We chose to assign thresholds obtained with targets contrast reversing at 8.5 Hz to the y-axis, while those with a target reversal rate of 17 Hz are on the x-axis. *Error bars* are 95% confidence intervals. *Empty symbols* with similar edge color and shapes are means of their matching subgroups. The overall mean is the *empty triangle* with a black edge. Data from Supplementary Table S1. Data collected from the authors are symbols with different face color.

Next, we examined the effect of contrast reversing both target and background, either at the same or different rates. There were two synchronous conditions, where the target and background reversed at 17 Hz (HH) or 8.5 Hz (LL), and two asynchronous conditions (HL and LH), where they differed. As shown in Figure 7A, mean thresholds were consistently lower for asynchronous than synchronous conditions but remained well matched within different synchronous and asynchronous rate pairings (HL = 0.438°; LH = 0.463°; HH = 0.520°; LL = 0.525°).

Pairwise contrasts confirmed this pattern. There were no significant differences between the synchronous conditions (LL–HH = 0.005°; *P* > 0.999, *t* = 0.587) or between asynchronous conditions (LH–HL = 0.025°; *P* = 0.608, *t* = 2.804). In contrast, comparisons between synchronous and asynchronous conditions sharing a common target or background frequency consistently favored the asynchronous condition: LL–LH = 0.061°; *P* < 0.001, *t* = 6.962; HL–LL = −0.086°; *P* < 0.001, *t* = −9.833; HL–HH = −0.081°; *P* < 0.001, *t* = −9.262; HH–LH = 0.056°; *P* < 0.001, *t* = 6.391. Pooling all asynchronous versus synchronous conditions yielded a significant threshold advantage for asynchronous presentation (0.071°; *P* < 0.001, *t* = 11.451).

Together, these results suggest that the key factor was the presence or absence of a mismatch in reversal rate between target and background, rather than the specific rate values. This can be confirmed via inspection of Figure 7B, where the same data are plotted in a way that allows comparison within synchronous and asynchronous conditions comprising different combinations of rate.

## Discussion

This study demonstrates that manipulations of image properties can significantly influence peripheral acuity in humans with normal vision. For targets presented on a uniform gray background, the main predictor of performance was the contrast polarity of the target. Superior acuity was found for static black targets compared to either white targets or targets that contrast reversed over time. However, a dramatically different pattern of results was found when targets were presented on a noise background. Here, dynamic contrast reversal produced significant improvements in performance. This held for situations where only the target was modulated, only the background was modulated, and both target and background were modulated at different rates.

The superiority of acuity for static black targets over static white targets was somewhat unexpected. In central vision, the ability to resolve two closely separated light squares presented on a dark background is approximately 64% *better* than when dark squares are presented on a light background.^19^ While most visual acuity letter charts use black letters on a white background, there are theoretical reasons why white letters on a black background might lead to improved visual performance.^20^ First, the change in background light level alters the calculated contrast of the targets. Second, light scatter and aberrations, which affect the quality of the retinal image, should be reduced for reversed polarity conditions, since less light enters the eye. Using Landolt C targets, Westheimer and collaborators^21^ confirmed that the minimum angle of resolution was significantly smaller for white letters on a dark background. Moreover, this difference was more pronounced in older participants, in whom light scatter is generally increased.^21^ Across a range of luminance levels, performance with white targets on dark backgrounds exceeds that for black targets on light backgrounds when natural ocular aberrations are present.^22^ This difference is reduced, but not eliminated, when aberrations are corrected.^22^ When letter contrast is equal and defined in Weber terms, the ability to detect letters of different contrast polarity (luminance increments versus decrements) is similar,^23^ suggesting contrast-dependent changes underlie performance differences when the backgrounds vary in luminance.

Our study differs in a couple of important ways. First, by using a gray background, the Weber contrast of black and white letter C targets was equated, and changes in overall luminance linked to target configurations were minimal. Second, whereas previous studies focused on acuity for foveated stimuli, our thresholds were measured at 10° in the peripheral field. At present, it is difficult to isolate which of these factors is critical for producing an advantage for black targets. One possible explanation may lie in the perceptual effect of “irradiation,” where the spatial extent of brighter areas appears increased relative to a darker area of equivalent size.^24^^–^^26^ In this situation, angular resolution may be impaired by the fact that the gap to be resolved to complete the task is increasingly filled by light for white letters, but not for black. To explain our results, this effect would need to have a stronger influence on stimuli presented in the periphery. Interestingly, in his *Treatise on Physiological Optics*, Helmholtz noted that strength of irradiation effects increases with retinal blur caused by accommodative error. Whether peripheral blur produces the same effect remains to be tested.

Spatial resolution limits, at least beyond the fovea, are broadly consistent with sampling constraints imposed by the dendritic fields of retinal ganglion cells.^27^^,^^28^ If there is any asymmetry in the spatial scale of pathways that represent targets defined by increments (ON-center cells) and decrements (OFF-center cells) in luminance, then differences in acuity for each target type may be observed. Anatomic studies of the human retina show that OFF-center cells form a higher density mosaic than their ON-center counterparts.^27^ This predicts that the spatial resolution of the OFF-center mosaic should be superior, especially for peripheral regions of the retina, where a difference in dendritic and receptive field size has been observed.^27^^,^^29^ This asymmetry between ON- and OFF-center ensembles is thought to underpin detection advantages for decremental stimuli measured psychophysically,^30^^–^^32^ but our data suggest this may also extend to acuity tasks.

Dynamically reversing the contrast polarity of the target produced differing effects depending on the characteristics of the background. On a uniform background, performance differences between target states were modest. In keeping with previous findings,^16^ thresholds with a contrast-reversing target tended to be better than those obtained with a static white target. However, performance did not exceed that observed with a static black target. This pattern of results changed dramatically when a static noise background was used. In this instance, polarity reversal of the target clearly enhanced acuity, relative to static black and static white target conditions. This effect can be understood in terms of the differential impact that adding noise had on acuity for static and dynamic targets. For static targets, the addition of the background noise substantially elevated thresholds. In contrast, thresholds for contrast-reversing targets presented on noise were equivalent, or even lower, than those obtained on a uniform background.

A potential explanation for the acuity benefit observed with dynamic contrast reversal of the target is that it promotes segmentation of the target from the noisy background. In support of this hypothesis, contrast reversing the background also improved performance for static targets, particularly at the higher reversal rate. Moreover, contrast modulating both target and background also resulted in systematic improvements in acuity, but only when there was a temporal mismatch between the two reversal rates. This indicates that it is not the presence or rate of contrast modulation that is critical per se, but rather the presence of a temporal cue that supports appropriate grouping and segmentation of target and background features.

It is well known that temporal information can be used effectively to group local image features for form judgments.^33^ Moreover, what seems to be important for temporal binding of spatial information is the pattern of changes over time, rather than the precise timing of the changes.^33^ If the temporal pattern is the factor that enhances spatial grouping, then it should not matter whether the background or target reverses faster, just that they are sufficiently different, and the difference is maintained. The temporal modulation of targets and backgrounds not only boosts spatial grouping processes but also acts as a powerful segmentation cue.^34^ The additional image segmentation that occurs for asynchronous conditions is lost when both background and target change synchronously. Previous work has shown that for effective figure-ground grouping and segmentation, a temporal offset of around 10 ms is required.^35^ The neural signals thought to mediate figure-ground processes develop rapidly in the visual cortex (50–300 ms) but are relatively transient.^36^ As such, frequently reintroducing the temporal signal is likely to reinstate and maintain the perceptual enhancement. Temporal modulations may therefore be increasingly effective in more complex visual tasks, where discrete elements need to be grouped and a global shape signal extracted, such as reading words or identifying a face in a crowd—particularly when this is performed against a complex spatially structured background.

While alternative mechanisms exist that could potentially explain the benefits of contrast modulation under some conditions, they fail to provide a complete characterization of the effects reported here. For example, improvements in performance following contrast reversal of a noisy background could readily be explained via a temporal summation mechanism, which acts to “average out” the noise signal over time. However, applying the same mechanism to a contrast-modulated target would dramatically reduce its effective contrast and likely impair performance. Adaptation to high temporal frequency flicker has also been shown to sharpen visual acuity.^37^ In principle, participants in the present study could have accumulated adaptation over repeated trials during an experimental session, which was blocked by condition. However, this explanation would predict that performance should be dependent on the absolute contrast-reversal rates of the stimuli, rather than the relative rates of target and background. Indeed, of the stimulus conditions tested, flickering both target and background at a fast rate should have been optimal for driving adaptation-based sharpening, yet this condition resulted in relatively poor acuity.

The results presented here suggest that adding temporal modulation, in a way that facilitates the grouping of features of an object and their segmentation from the rest of the scene, is likely to aid performance on acuity-limited tasks such as discriminating between faces or differentiating between small objects of interest (e.g., coins or keys). The ability to extract spatial form information using temporal cues can be significantly enhanced with perceptual learning^38^ and is therefore a good candidate for training once a stable PRL is established in patients with central vision loss. Display systems that can hold an object of interest at the PRL and selectively modulate it would be particularly well suited for this purpose. There are now several extended reality systems available that would have the eye-tracking and image-rendering capabilities necessary to achieve this. An additional consideration arises from the fact that patients with macular disease often show abnormal patterns of eye movements (e.g., increased microsaccade amplitude and drift) when attempting to fixate.^39^^–^^41^ This introduces image motion that varies in direction and speed and is thought to play a contributory role in the visual loss these patients experience.^41^^–^^43^ In previous work, we have shown that for both static and dynamic targets that are contrast reversed, improvements in acuity thresholds are relatively robust to the introduction of motion that mimics greatly amplified fixational eye movements.^16^ Therefore, the gains that arise from contrast modulation do not require high levels of positional stability and should be realizable even in patients with abnormal fixation patterns. Adding temporal modulation can also reduce the effects of crowding in the peripheral field.^44^ This may have implications for reading performance in the periphery, where adjacent characters and words can crowd one another, limiting both reading speed and acuity. In the future, it will be important to determine whether adding temporal modulation to images acts in isolation or can be combined with other image-enhancing approaches, such as edge enhancement algorithms, to improve visual performance in the peripheral field.

## Supplementary Material

Supplement 1
