# Supplementary material for: The Effect of Contrast Reversal on Peripheral Visual Acuity
Source: Transl Vis Sci Technol. 2025 Aug 19;14(8):23. doi: 10.1167/tvst.14.8.23 (PMC12369918; doi:10.1167/tvst.14.8.23)
Supplement: Supplement 1 [file tvst-14-8-23_s001.pdf]

The Effects of Contrast Reversal on Peripheral Visual Acuity – supplementary material

This document contains the statistical analyses conducted to support the manuscript:

“The Effects of Contrast Reversal on Peripheral Visual Acuity”

The companion R notebook from which this document was generated is available upon request. Several commands have been commented out (#), to produce a readable document. If their execution is desired, please remove the commenting in the R notebook.

To begin, we need to install and load several R packages used in the analyses:

```
# the following command will install required packages if not installed
#install.packages(c('lme4','Matrix','lmerTest','emmeans','tidyverse','easysta
ts','report','strengejacke','rstatix'))
# the following commands will make active the required packages
library(lme4)
library(lmerTest)
library(emmeans)
library(report)
library(tidyverse)
library(easystats)
library(rstatix)
library(strengejacke)
library(flexdashboard)
library(finalfit)
# the following command reports all packages in use
report_packages()
```

```
##   - flexdashboard (version 0.6.2; Aden-Buie G et al., 2023)
##   - lme4 (version 1.1.35.5; Bates D et al., 2015)
##   - Matrix (version 1.7.1; Bates D et al., 2024)
##   - effectsize (version 0.8.9; Ben-Shachar MS et al., 2020)
##   - lubridate (version 1.9.3; Grolemund G, Wickham H, 2011)
##   - finalfit (version 1.0.8; Harrison E et al., 2024)
##   - rstatix (version 0.7.2; Kassambara A, 2023)
##   - lmerTest (version 3.1.3; Kuznetsova A et al., 2017)
##   - emmeans (version 1.10.5; Lenth R, 2024)
##   - ggeffects (version 2.0.0; Lüdecke D, 2018)
##   - sjmisc (version 2.8.10; Lüdecke D, 2018)
##   - esc (version 0.5.1; Lüdecke D, 2019)
##   - strengejacke (version 0.5.0; Lüdecke D, 2019)
##   - sjlabelled (version 1.2.0; Lüdecke D, 2022)
##   - sjPlot (version 2.8.17; Lüdecke D, 2024)
##   - sjstats (version 0.19.0; Lüdecke D, 2024)
```

```
##   - parameters (version 0.24.0; Lüdecke D et al., 2020)
##   - performance (version 0.12.4; Lüdecke D et al., 2021)
##   - easystats (version 0.7.3; Lüdecke D et al., 2022)
##   - see (version 0.9.0; Lüdecke D et al., 2021)
##   - insight (version 1.0.0; Lüdecke D et al., 2019)
##   - bayestestR (version 0.15.0; Makowski D et al., 2019)
##   - modelbased (version 0.8.9; Makowski D et al., 2020)
##   - report (version 0.5.9; Makowski D et al., 2023)
##   - correlation (version 0.8.6; Makowski D et al., 2022)
##   - tibble (version 3.2.1; Müller K, Wickham H, 2023)
##   - datawizard (version 0.13.0; Patil I et al., 2022)
##   - R (version 4.4.2; R Core Team, 2024)
##   - ggplot2 (version 3.5.1; Wickham H, 2016)
##   - forcats (version 1.0.0; Wickham H, 2023)
##   - stringr (version 1.5.1; Wickham H, 2023)
##   - tidyverse (version 2.0.0; Wickham H et al., 2019)
##   - dplyr (version 1.1.4; Wickham H et al., 2023)
##   - purrr (version 1.0.2; Wickham H, Henry L, 2023)
##   - readr (version 2.1.5; Wickham H et al., 2024)
##   - tidyr (version 1.3.1; Wickham H et al., 2024)
```

Load the main dataset and name it revDS, it contains all reversals from all runs and will be the base of further analyses. We also define the factors to set up the linear model analysis

```
revDS <- read_delim('revDS.txt')

## Rows: 5886 Columns: 6
## ── Column specification
────────────────────────────────────────────────────────

## Delimiter: ","
## chr (2): T, B
## dbl (4): ID, Cd, Rn, rev
##
## ℹ Use `spec()` to retrieve the full column specification for this data.
## ℹ Specify the column types or set `show_col_types = FALSE` to quiet this
message.

revDS$ID <- as.factor(revDS$ID)
revDS$Cd <- as.factor(revDS$Cd)
revDS$T <- as.factor(revDS$T)
revDS$B <- as.factor(revDS$B)
revDS$Rn <- as.factor(revDS$Rn)
```

Next we compute various summary statistics. First, we take the average of the last six reversals for each staircase (Rn) performed by each participant (ID) in each condition (determined by T,B).

```
revDS_Rn <- revDS |>
    group_by(ID,T,B,Cd,Rn) |>
    get_summary_stats(rev, show = c("mean","ci","sd","se"))
#revDS_Rn
```

We also summarise the thresholds per participant (ID), target (T) and background (B) state

```
# this generates the data for Table S1 and Figures 3 to 7

revDS_Id <- revDS |>
  group_by(ID,T,B,Cd) |>
  get_summary_stats(rev, show = c("mean","ci","sd","se"))
#revDS_Id
```

Finally the population data by target (T) and background (B) state, which characterize each condition and become the fixed effects of the linear model. This summary statistics is plotted in Fig 2 and cited in the manuscript as Table S3

```
# this produces Table S2 and data shown in Figure 2

revDS_Cd <- revDS |>
  group_by(T,B,Cd) |>
  get_summary_stats(rev, show = c("mean","ci","sd","se"))
revDS_Cd

## # A tibble: 16 × 9
##    Cd    T     B     variable     n  mean    ci    sd    se
##    <fct> <fct> <fct> <fct>    <dbl> <dbl> <dbl> <dbl> <dbl>
##  1 7     h     d     rev        324 0.438 0.014 0.128 0.007
##  2 1     h     h     rev        360 0.52  0.015 0.149 0.008
##  3 2     h     n     rev        360 0.495 0.014 0.14  0.007
##  4 3     h     u     rev        372 0.476 0.013 0.124 0.006
##  5 4     l     d     rev        360 0.525 0.018 0.176 0.009
##  6 8     l     h     rev        324 0.463 0.018 0.165 0.009
##  7 5     l     n     rev        360 0.457 0.015 0.146 0.008
##  8 6     l     u     rev        378 0.464 0.013 0.132 0.007
##  9 10    s     d     rev        360 0.532 0.018 0.176 0.009
## 10 9     s     h     rev        384 0.475 0.013 0.132 0.007
## 11 11    s     n     rev        360 0.552 0.022 0.208 0.011
## 12 12    s     u     rev        360 0.459 0.013 0.128 0.007
## 13 14    w     d     rev        396 0.602 0.018 0.186 0.009
## 14 13    w     h     rev        396 0.581 0.017 0.172 0.009
## 15 15    w     n     rev        396 0.602 0.019 0.19  0.01
## 16 16    w     u     rev        396 0.506 0.013 0.127 0.006
```

We run the linear model on the averages of the last six reversal of each staircase, this is the dataset revDS_Rn, we add random effects to the participant Id factor

```
# this is the main linear mixed model used for analyses

rm1 <- lmer(rev ~ T*B + (1|ID/Rn), data = revDS, REML = F)
#summary(rm1)
```

We can get the model parameters in a table (cited as Table S3 in the main manuscript)

```
parameters(rm1,standardise=T)
```

```
## # Fixed Effects
##
## Parameter     | Coefficient |       SE |         95% CI | t(5867) |      p
## --------------------------------------------------------------------------
## (Intercept)   |        0.44 |     0.03 | [ 0.38,  0.51] |   13.19 | < .001
## T [l]         |        0.09 | 8.75e-03 | [ 0.07,  0.10] |    9.85 | < .001
## T [s]         |        0.09 | 8.75e-03 | [ 0.08,  0.11] |   10.58 | < .001
## T [w]         |        0.16 | 8.59e-03 | [ 0.15,  0.18] |   19.16 | < .001
## B [h]         |        0.08 | 8.75e-03 | [ 0.06,  0.10] |    9.27 | < .001
## B [n]         |        0.06 | 8.75e-03 | [ 0.04,  0.07] |    6.46 | < .001
## B [u]         |        0.04 | 8.69e-03 | [ 0.03,  0.06] |    5.05 | < .001
## T [l] × B [h] |       -0.14 |     0.01 | [-0.17, -0.12] |  -11.47 | < .001
## T [s] × B [h] |       -0.12 |     0.01 | [-0.15, -0.10] |  -10.16 | < .001
## T [w] × B [h] |       -0.10 |     0.01 | [-0.12, -0.08] |   -8.50 | < .001
## T [l] × B [n] |       -0.12 |     0.01 | [-0.15, -0.10] |  -10.23 | < .001
## T [s] × B [n] |       -0.04 |     0.01 | [-0.06, -0.01] |   -2.99 | 0.003 
## T [w] × B [n] |       -0.06 |     0.01 | [-0.08, -0.03] |   -4.74 | < .001
## T [l] × B [u] |       -0.09 |     0.01 | [-0.12, -0.07] |   -7.78 | < .001
## T [s] × B [u] |       -0.12 |     0.01 | [-0.14, -0.09] |   -9.58 | < .001
## T [w] × B [u] |       -0.14 |     0.01 | [-0.16, -0.12] |  -11.77 | < .001
##
## # Random Effects
##
## Parameter               | Coefficient
## -------------------------------------
## SD (Intercept: Rn:ID) |        0.02
## SD (Intercept: ID)    |        0.10
## SD (Residual)         |        0.11

##
## Uncertainty intervals (equal-tailed) and p-values (two-tailed) computed
##   using a Wald t-distribution approximation.
```

Using the fitted statistical model we can now estimate means from the model with

```
# these are the marginal means estimated by the model, can be compared with
the data actually recorded (displayed in Figure 2)

estimate_means(rm1, by=c("T","B"), pbkrtest.limit = 6000, lmerTest.limit =
6000)

## Estimated Marginal Means
##
## T | B | Mean |   SE |       95% CI
## ----------------------------------
## h | d | 0.44 | 0.04 | [0.36, 0.52]
## l | d | 0.53 | 0.04 | [0.45, 0.60]
## s | d | 0.53 | 0.04 | [0.46, 0.61]
## w | d | 0.61 | 0.04 | [0.53, 0.68]
## h | h | 0.52 | 0.04 | [0.45, 0.60]
## l | h | 0.47 | 0.04 | [0.39, 0.54]
```

```
## s | h | 0.49 | 0.04 | [0.41, 0.57]
## w | h | 0.59 | 0.04 | [0.51, 0.66]
## h | n | 0.50 | 0.04 | [0.42, 0.57]
## l | n | 0.46 | 0.04 | [0.38, 0.54]
## s | n | 0.55 | 0.04 | [0.48, 0.63]
## w | n | 0.61 | 0.04 | [0.53, 0.68]
## h | u | 0.48 | 0.04 | [0.41, 0.56]
## l | u | 0.48 | 0.04 | [0.40, 0.55]
## s | u | 0.46 | 0.04 | [0.38, 0.54]
## w | u | 0.51 | 0.04 | [0.43, 0.59]
##
## Marginal means estimated at T
```

and compare to the actual values in the data. Further, using the model we can predict the response variable used in the model, this is, estimates of the entries of the dataset that we are using and compare to the data with:

```
# if uncommented the command below will produce expectation values of the model for each reversal (long list)

expect_rm1 <- estimate_expectation(rm1)
#expect_rm1
```

Or, for individual predictions (note the difference in the CI):

```
# if uncommented the command below will provide sample predictions of the reversals base on the distributions fitted to the data (long list)

predct_rm1 <- estimate_prediction(rm1)
#predict_rm1
```

Now we compute the contrasts for the model using T and B as factors

```
# the command below will generate Table S4 containing all 120 pairwise contrasts Bonferroni-corrected (without the highlight); if uncommented it will show the table in the notebook and R console

emm_options(pbkrtest.limit=6000,lmerTest.limit=6000)
rm1_bonf <- estimate_contrasts(rm1,"T*B",p_adjust = "bonferroni")
#rm1_bonf
```

And extract those contrasts that are significant

```
rm1_bf_sig <- rm1_bonf[rm1_bonf[,9]<0.05,]
#rm1_bf_sig
```

Contrasts:

besides taking contrast pairwise over all combinations of states of target and background, we shall use customised contrasts to address the question raised in the manuscript

First we need to compute the marginal means grid

```r
emmrm1 <- emmeans(rm1, ~ T*B)
```

customised contrasts can be obtain addressing the specific comparison (building the matrix):

examining the effect of B on T and of T on B we get:

```r
contrast(emmrm1,simple = "each", adjust = "bonferroni")

## $`simple contrasts for T`
## B = d:
##  contrast  estimate      SE   df t.ratio p.value
##  h effect -0.085816 0.00542 5841 -15.824  <.0001
##  l effect  0.000329 0.00522 5840   0.063  1.0000
##  s effect  0.006787 0.00522 5840   1.300  0.7747
##  w effect  0.078700 0.00508 5880  15.479  <.0001
##
## B = h:
##  contrast  estimate      SE   df t.ratio p.value
##  h effect  0.005846 0.00521 5848   1.122  1.0000
##  l effect -0.050144 0.00541 5849  -9.266  <.0001
##  s effect -0.024716 0.00512 5888  -4.828  <.0001
##  w effect  0.069015 0.00507 5884  13.604  <.0001
##
## B = n:
##  contrast  estimate      SE   df t.ratio p.value
##  h effect -0.031348 0.00520 5840  -6.034  <.0001
##  l effect -0.070028 0.00520 5840 -13.480  <.0001
##  s effect  0.024763 0.00520 5840   4.767  <.0001
##  w effect  0.076613 0.00506 5880  15.146  <.0001
##
## B = u:
##  contrast  estimate      SE   df t.ratio p.value
##  h effect  0.001764 0.00512 5854   0.344  1.0000
##  l effect -0.006056 0.00510 5867  -1.187  0.9416
##  s effect -0.022084 0.00518 5854  -4.261  0.0001
##  w effect  0.026375 0.00505 5886   5.228  <.0001
##
## Degrees-of-freedom method: kenward-roger
## P value adjustment: bonferroni method for 4 tests
##
## $`simple contrasts for B`
## T = h:
##  contrast estimate      SE   df t.ratio p.value
##  d effect -0.04538 0.00543 5837  -8.352  <.0001
##  h effect  0.03577 0.00523 5837   6.836  <.0001
##  n effect  0.01111 0.00523 5837   2.124  0.1348
##  u effect -0.00150 0.00518 5860  -0.289  1.0000
##
## T = l:
```

```
##  contrast estimate      SE   df t.ratio p.value
##  d effect  0.04485 0.00523 5839   8.577  <.0001
##  h effect -0.01614 0.00543 5839  -2.972  0.0119
##  n effect -0.02348 0.00523 5839  -4.491  <.0001
##  u effect -0.00523 0.00516 5874  -1.014  1.0000
##
## T = s:
##  contrast estimate      SE   df t.ratio p.value
##  d effect  0.02365 0.00520 5842   4.547  <.0001
##  h effect -0.01837 0.00511 5886  -3.595  0.0013
##  n effect  0.04365 0.00520 5842   8.393  <.0001
##  u effect -0.04892 0.00520 5842  -9.407  <.0001
##
## T = w:
##  contrast estimate      SE   df t.ratio p.value
##  d effect  0.02907 0.00497 5834   5.850  <.0001
##  h effect  0.00887 0.00497 5834   1.785  0.2973
##  n effect  0.02901 0.00497 5834   5.838  <.0001
##  u effect -0.06695 0.00497 5834 -13.473  <.0001
##
## Degrees-of-freedom method: kenward-roger
## P value adjustment: bonferroni method for 4 tests

# the values above give us the effects of one factor at fixed levels of the
companion factor
```

We have used above Bonferroni correction for multiple tests, arguably the most conservative correction. The ‘estimates’ assess the discrepancy of the levels of the factor considered with respect to the mean, taken at a fix level of the other factor. In terms of Figure 2 in the manuscript, this means exploring the modulation of the T factor along each of the backgrounds separately (each of the lines plotted in the figure), as well as exploring the modulation across levels of B along each type of target held fixed (verticals taken at each abscissa point in Figure 2). Note that these are not ‘pairwise’ contrasts, the reference used is the mean across all level of the factor being considered. Loosely speaking this are estimates of the main effect of a factor at a fixed level of the other.

The emmgrid object emmrm1 has been computed including the interactions. When this is done, both factors are combined so that the first factor varies first through all its levels keeping the level of the second factor fix and so on. This allows the construction of ‘matrices’ or lists to explore contrasts in specific situations.

For example, to compute contrasts among the conditions in which both target and background remained static, a custom ‘matrix’ for this family can be defined:

```
staticgroup <- list(`BN - BU`=c(0,0,0,0,0,0,0,0,0,0,1,0,0,0,-1,0), # by tgt B
                    `WN - WU`  = c(0,0,0,0,0,0,0,0,0,0,0,1,0,0,0,-1), # by tgt W
                    `WU - BU`  = c(0,0,0,0,0,0,0,0,0,0,0,0,0,0,-1,1), # by bkg U
                    `WN - BN`  = c(0,0,0,0,0,0,0,0,0,0,-1,1,0,0,0,0), # by bkg N
                    `WN - BU`  = c(0,0,0,0,0,0,0,0,0,0,0,1,0,0,-1,0), # BU vs WN
```

```r
                 `WU - BN` = c(0,0,0,0,0,0,0,0,0,0,-1,0,0,0,0,1)) # WU vs BN

stat_bf <- contrast(emmrm1,staticgroup,adjust = "bonferroni")
print(stat_bf)
```

```
##  contrast estimate      SE   df t.ratio p.value
##  BN - BU    0.0926 0.00851 5834  10.877  <.0001
##  WN - WU    0.0960 0.00811 5834  11.825  <.0001
##  WU - BU    0.0485 0.00834 5868   5.807  <.0001
##  WN - BN    0.0518 0.00834 5868   6.214  <.0001
##  WN - BU    0.1444 0.00834 5868  17.307  <.0001
##  WU - BN   -0.0441 0.00834 5868  -5.286  <.0001
##
## Degrees-of-freedom method: kenward-roger
## P value adjustment: bonferroni method for 6 tests
```

The values indicate highly significant differences among the levels conforming to the group, consistent with Figure 2 in the Results section. Note that the correction for multiple tests only considers the contrasts used, therefore, while the estimates and t.ratios do not change with respect to those shown on Table S4, the p-values do. All contrasts reported with the figures in the main manuscript were taken from Table S4, therefore, corresponding to the most conservative assessment of significance.

For the fully dynamic group where both targets and backgrounds contrast reverse, we need:

```r
dynall  <-  list(`HH - LL` = c(0,-1,0,0,1,0,0,0,0,0,0,0,0,0,0,0), # sync cds
                 `LH - HL` = c(-1,0,0,0,0,1,0,0,0,0,0,0,0,0,0,0), # async cds
                 `LL - LH` = c(0,1,0,0,0,-1,0,0,0,0,0,0,0,0,0,0), # by tgt L
                 `LL - HL` = c(-1,1,0,0,0,0,0,0,0,0,0,0,0,0,0,0), # by bkg L
                 `HH - HL` = c(-1,0,0,0,1,0,0,0,0,0,0,0,0,0,0,0), # by tgt H
                 `HH - LH` = c(0,0,0,0,1,-1,0,0,0,0,0,0,0,0,0,0)) # by bkg H
dynall_bf <- contrast(emmrm1,dynall, adjust = "bonferroni")
print(dynall_bf)
```

```
##  contrast estimate      SE   df t.ratio p.value
##  HH - LL   -0.0050 0.00851 5834  -0.587  1.0000
##  LH - HL    0.0252 0.00897 5834   2.804  0.0304
##  LL - LH    0.0610 0.00876 5834   6.962  <.0001
##  LL - HL    0.0861 0.00876 5834   9.833  <.0001
##  HH - HL    0.0811 0.00876 5834   9.262  <.0001
##  HH - LH    0.0560 0.00876 5834   6.391  <.0001
##
## Degrees-of-freedom method: kenward-roger
## P value adjustment: bonferroni method for 6 tests
```

This set of contrasts corresponds to Figure 7 in the main manuscript. It indicates that there are no significant differences between the two synchronous conditions (HH - LL), and just significant between the two asynchronous conditions (LH - HL), with HL being the lowest overall. There are strong differences between each of the asynchronous conditions (HL,

LH) and both synchronous conditions (HH, LL). Contrasts have been arranged so that the estimate of the difference in means is always positive.

To complete the description of the four subgroups mentioned in the paper we need the case where targets were static on backgrounds reversing contrast and where target reversed contrast on static backgrounds. We do that next.

```
stbackgCRtgt <- list(`HU - LU` = c(0,0,0,0,0,0,0,0,0,0,0,0,1,-1,0,0), # on
bkg U
                 `HN - LN` = c(0,0,0,0,0,0,0,0,1,-1,0,0,0,0,0,0), # on bkg N
                 `HN - HU` = c(0,0,0,0,0,0,0,0,1,0,0,0,-1,0,0,0), # by tgt H
                 `LU - LN` = c(0,0,0,0,0,0,0,0,0,-1,0,0,0,1,0,0), # by tgt L
                 `HU - LN` = c(0,0,0,0,0,0,0,0,0,-1,0,0,1,0,0,0),  # HU vs LN
                 `HN - LU` = c(0,0,0,0,0,0,0,0,1,0,0,0,0,-1,0,0))  # HN vs LU
acrbkg <- list(`bkgU - bkgN` = c(0,0,0,0,0,0,0,0,-1/2,-1/2,0,0,1/2,1/2,0,0))
acrfreq <- list(`tgtH - tgtL` = c(0,0,0,0,0,0,0,0,1/2,-1/2,0,0,1/2,-1/2,0,0))
contrast(emmrm1,stbackgCRtgt,adjust = "bonferroni")

##  contrast estimate      SE   df t.ratio p.value
##  HU - LU   0.00782 0.00834 5846   0.937  1.0000
##  HN - LN   0.03868 0.00851 5834   4.545  <.0001
##  HN - HU   0.01261 0.00845 5852   1.492  0.8144
##  LU - LN   0.01825 0.00842 5862   2.166  0.1820
##  HU - LN   0.02607 0.00845 5852   3.085  0.0123
##  HN - LU   0.02043 0.00842 5862   2.425  0.0920
##
## Degrees-of-freedom method: kenward-roger
## P value adjustment: bonferroni method for 6 tests

contrast(emmrm1,acrbkg)

##  contrast    estimate      SE   df t.ratio p.value
##  bkgU - bkgN  0.00282 0.00597 5871   0.472  0.6369
##
## Degrees-of-freedom method: kenward-roger

contrast(emmrm1,acrfreq)

##  contrast    estimate      SE   df t.ratio p.value
##  tgtH - tgtL   0.0233 0.00596 5840   3.901  0.0001
##
## Degrees-of-freedom method: kenward-roger
```

There are no significant differences between contrast reversing targets at H or L rate on uniform background; this difference becomes significant on a static patterned background (N). Contrasting the conditions pooled by the type of background does not show significance, while pooling the conditions by the rate of target contrast reversal shows that the means differ significantly, both predictions consistent with Figure 2.

The last group is that of static targets on contrast reversing backgrounds

```r
sttgtCRbackg <- list(`WH - BH` = c (0,0,0,0,0,0,-1,1,0,0,0,0,0,0,0,0),# bkg H
                  `WL - BL` = c(0,0,-1,1,0,0,0,0,0,0,0,0,0,0,0,0), # bkg L
                  `BL - BH` = c(0,0,1,0,0,0,-1,0,0,0,0,0,0,0,0,0), # tgt B
                  `WL - WH` = c(0,0,0,1,0,0,0,-1,0,0,0,0,0,0,0,0),  # tgt W
                  `WL - BH` = c(0,0,0,1,0,0,-1,0,0,0,0,0,0,0,0,0),  # WL vs BH
                  `WH - BL` = c(0,0,-1,0,0,0,0,1,0,0,0,0,0,0,0,0))  # WH vs BL

contrast(emmrm1,sttgtCRbackg,adjust = 'bonferroni')

##  contrast estimate      SE   df t.ratio p.value
##  WH - BH    0.0937 0.00823 5890  11.385  <.0001
##  WL - BL    0.0719 0.00834 5868   8.618  <.0001
##  BL - BH    0.0420 0.00840 5874   5.002  <.0001
##  WL - WH    0.0202 0.00811 5834   2.490  0.0769
##  WL - BH    0.1139 0.00823 5890  13.839  <.0001
##  WH - BL    0.0517 0.00834 5868   6.197  <.0001
##
## Degrees-of-freedom method: kenward-roger
## P value adjustment: bonferroni method for 6 tests

# above: there are no significant differences between conditions where a
static white target is presented on backgrounds reversing contrasts at H and
L rate (the two closest points along the vertical at abscissa point 'W' on
Figure 2). All other comparisons are significant

# when conditions are pooled by type of background or by target colour, means
are significantly different:

# pooled across background:

acrCRbkg <- list(`bkgL-bkgH` = c(0,0,1/2,1/2,0,0,-1/2,-1/2,0,0,0,0,0,0,0,0))
# pooled across targets:

acrtgcol <- list(`tgtW-tgtB` = c(0,0,-1/2,1/2,0,0,-1/2,1/2,0,0,0,0,0,0,0,0))
contrast(emmrm1,acrCRbkg)

##  contrast    estimate      SE   df t.ratio p.value
##  bkgL - bkgH   0.0311 0.00584 5858   5.327  <.0001
##
## Degrees-of-freedom method: kenward-roger

contrast(emmrm1,acrtgcol)

##  contrast    estimate      SE   df t.ratio p.value
##  tgtW - tgtB   0.0828 0.00588 5891  14.080  <.0001
##
## Degrees-of-freedom method: kenward-roger
```

Below we pooled together all contrasts in all four groups. p-values here differ from those in Table S4 due to the different number of comparisons.

```
allgroups <- contrast(emmrm1,c(staticgroup,stbackgCRtgt,sttgtCRbackg,dynall), adjust = 'bonferroni')
allgroups
```

```
##  contrast estimate      SE   df t.ratio p.value
##  BN - BU   0.09257 0.00851 5834  10.877  <.0001
##  WN - WU   0.09596 0.00811 5834  11.825  <.0001
##  WU - BU   0.04846 0.00834 5868   5.807  <.0001
##  WN - BN   0.05185 0.00834 5868   6.214  <.0001
##  WN - BU   0.14442 0.00834 5868  17.307  <.0001
##  WU - BN  -0.04411 0.00834 5868  -5.286  <.0001
##  HU - LU   0.00782 0.00834 5846   0.937  1.0000
##  HN - LN   0.03868 0.00851 5834   4.545  0.0001
##  HN - HU   0.01261 0.00845 5852   1.492  1.0000
##  LU - LN   0.01825 0.00842 5862   2.166  0.7280
##  HU - LN   0.02607 0.00845 5852   3.085  0.0491
##  HN - LU   0.02043 0.00842 5862   2.425  0.3682
##  WH - BH   0.09373 0.00823 5890  11.385  <.0001
##  WL - BL   0.07191 0.00834 5868   8.618  <.0001
##  BL - BH   0.04202 0.00840 5874   5.002  <.0001
##  WL - WH   0.02020 0.00811 5834   2.490  0.3077
##  WL - BH   0.11393 0.00823 5890  13.839  <.0001
##  WH - BL   0.05171 0.00834 5868   6.197  <.0001
##  HH - LL  -0.00500 0.00851 5834  -0.587  1.0000
##  LH - HL   0.02515 0.00897 5834   2.804  0.1216
##  LL - LH   0.06099 0.00876 5834   6.962  <.0001
##  LL - HL   0.08614 0.00876 5834   9.833  <.0001
##  HH - HL   0.08114 0.00876 5834   9.262  <.0001
##  HH - LH   0.05599 0.00876 5834   6.391  <.0001
##
## Degrees-of-freedom method: kenward-roger
## P value adjustment: bonferroni method for 24 tests
```

If, instead, we are interesting in assessing the modulation on each type of background separately, we have: Along the uniform background (U):

```
bckgroundU  <-  list(`LU - BU` = c(0,0,0,0,0,0,0,0,0,0,0,0,0,1,-1,0), # L - B
                 `HU - BU` = c (0,0,0,0,0,0,0,0,0,0,0,0,1,0,-1,0), # H - B
                 `WU - LU` = c(0,0,0,0,0,0,0,0,0,0,0,0,0,-1,0,1),  # W - L
                 `WU - HU` = c(0,0,0,0,0,0,0,0,0,0,0,0,-1,0,0,1),  # W - H
                 `WU - BU` = c(0,0,0,0,0,0,0,0,0,0,0,0,0,0,-1,1), # W - B
                 `LU - HU` = c(0,0,0,0,0,0,0,0,0,0,0,0,-1,1,0,0))  # L - H
contrast(emmrm1,bckgroundU,adjust = 'bonferroni')
```

```
##  contrast estimate      SE   df t.ratio p.value
##  LU - BU   0.01603 0.00842 5862   1.902  0.3430
##  HU - BU   0.02385 0.00845 5852   2.822  0.0288
##  WU - LU   0.03243 0.00826 5885   3.928  0.0005
##  WU - HU   0.02461 0.00828 5880   2.971  0.0179
##  WU - BU   0.04846 0.00834 5868   5.807  <.0001
##  LU - HU  -0.00782 0.00834 5846  -0.937  1.0000
```

```
##
## Degrees-of-freedom method: kenward-roger
## P value adjustment: bonferroni method for 6 tests
```

Note that the pairwise significance of these contrasts are different and enhanced with respect to what is reported in the subsection of Figure 4. The reason is that the values reported in the manuscript have been Bonferroni adjusted over the entire set of pairwise comparisons, while here we are only considering six level combinations because we are tracking only along the uniform background. While differences estimates and t-ratios do not change, the p-values do because the number of tests is much lower. Therefore, here the black target condition shows significant advantage over the contrast reversing target condition at high rate, and white target conditions show significantly higher means than the contrast reversing target condition at both rates tested. In spite of the changes in p-values, the effects reported and the conclusions reached remain the same.

For the static patterned background:

```
bckgroundN  <- list(`BN - LN` = c (0,0,0,0,0,0,0,0,0,-1,1,0,0,0,0,0), # H - B
                 `BN - HN` = c(0,0,0,0,0,0,0,0,-1,0,1,0,0,0,0,0), # L - B
                 `WN - LN` = c(0,0,0,0,0,0,0,0,0,-1,0,1,0,0,0,0), # W - B
                 `WN - HN` = c(0,0,0,0,0,0,0,0,-1,0,0,1,0,0,0,0),  # W - H
                 `WN - BN` = c(0,0,0,0,0,0,0,0,0,0,-1,1,0,0,0,0),  # W - L
                 `HN - LN` = c(0,0,0,0,0,0,0,0,1,-1,0,0,0,0,0,0))  # L - H
contrast(emmrm1,bckgroundN,adjust = 'bonferroni')
```

```
##  contrast estimate      SE   df t.ratio p.value
##  BN - LN    0.0948 0.00851 5834  11.138  <.0001
##  BN - HN    0.0561 0.00851 5834   6.593  <.0001
##  WN - LN    0.1466 0.00834 5868  17.573  <.0001
##  WN - HN    0.1080 0.00834 5868  12.938  <.0001
##  WN - BN    0.0518 0.00834 5868   6.214  <.0001
##  HN - LN    0.0387 0.00851 5834   4.545  <.0001
##
## Degrees-of-freedom method: kenward-roger
## P value adjustment: bonferroni method for 6 tests
```

Similar considerations to those made for the uniform background (U) in relation to Figure 4, apply to the values for tests on a noise background (N) with respect to the reported with Figure 5. Bonferroni correction done on the entire set of pairwise contrasts is per force more conservative than when applied to an isolated background in spite of the invariance in difference estimates and t-ratios.

For the backgrounds contrast reversing at low (L) and (H) rate we have, respectively:

```
bckgroundL  <- list(`WL - BL` = c (0,0,-1,1,0,0,0,0,0,0,0,0,0,0,0,0), # H - B
                 `WL - LL` = c(0,-1,0,1,0,0,0,0,0,0,0,0,0,0,0,0), # L - B
                 `WL - HL` = c(-1,0,0,1,0,0,0,0,0,0,0,0,0,0,0,0), # W - B
                 `BL - HL` = c(-1,0,1,0,0,0,0,0,0,0,0,0,0,0,0,0),  # W - H
                 `BL - LL` = c(0,-1,1,0,0,0,0,0,0,0,0,0,0,0,0,0),  # W - L
```

```
                 `LL - HL` = c(-1,1,0,0,0,0,0,0,0,0,0,0,0,0,0,0))  # L - H
contrast(emmrm1,bckgroundL, adjust = 'bonferroni')

##  contrast estimate      SE   df t.ratio p.value
##  WL - BL   0.07191 0.00834 5868   8.618  <.0001
##  WL - LL   0.07837 0.00834 5868   9.392  <.0001
##  WL - HL   0.16452 0.00860 5868  19.135  <.0001
##  BL - HL   0.09260 0.00876 5834  10.570  <.0001
##  BL - LL   0.00646 0.00851 5834   0.759  1.0000
##  LL - HL   0.08614 0.00876 5834   9.833  <.0001
##
## Degrees-of-freedom method: kenward-roger
## P value adjustment: bonferroni method for 6 tests

bckgroundH  <- list(`BH - LH` = c(0,0,0,0,0,-1,1,0,0,0,0,0,0,0,0,0),  # W - H
                    `BH - HH` = c(0,0,0,0,-1,0,1,0,0,0,0,0,0,0,0,0),  # W - L
                    `WH - LH` = c(0,0,0,0,0,-1,0,1,0,0,0,0,0,0,0,0), # W - B
                    `WH - HH` = c(0,0,0,0,-1,0,0,1,0,0,0,0,0,0,0,0), # L - B
                    `WH - BH` = c (0,0,0,0,0,0,-1,1,0,0,0,0,0,0,0,0), # H - B
                    `HH - LH` = c(0,0,0,0,1,-1,0,0,0,0,0,0,0,0,0,0))  # L - H
contrast(emmrm1,bckgroundH, adjust = 'bonferroni')

##  contrast estimate      SE   df t.ratio p.value
##  BH - LH    0.0254 0.00865 5874   2.939  0.0199
##  BH - HH   -0.0306 0.00840 5874  -3.638  0.0017
##  WH - LH    0.1192 0.00860 5868  13.859  <.0001
##  WH - HH    0.0632 0.00834 5868   7.570  <.0001
##  WH - BH    0.0937 0.00823 5890  11.385  <.0001
##  HH - LH    0.0560 0.00876 5834   6.391  <.0001
##
## Degrees-of-freedom method: kenward-roger
## P value adjustment: bonferroni method for 6 tests
```

Or, combining all data taken on contrast reversing backgrounds, regardless of the rate:

```
all_bckgd <-
contrast(emmrm1,c(bckgroundH,bckgroundL,bckgroundN,bckgroundU),adjust =
'bonferroni')
```

It can be checked that these estimates based on the model match the data in Figure 2.

To obtain the group contrasts reported in each of figures 3 to 7 in the Results we follow similar procedure:

```
# group contrasts for Figure 3
figure3A <- list(`Fig3A: W_UN - B_UN` = c(0,0,0,0,0,0,0,0,0,0,-1/2,1/2,0,0,-
1/2,1/2))
figure3B <- list(`Fig3B: W_HL - B_HL` = c(0,0,-1/2,1/2,0,0,-
1/2,1/2,0,0,0,0,0,0,0,0))
figure3_all <- list(`Fig3: W_all - B_all` = c(0,0,-1/4,1/4,0,0,-1/4,1/4,0,0,-
1/4,1/4,0,0,-1/4,1/4))
```

```
#contrast(emmrm1,c(figure3A,figure3B,figure3_all), adjust = 'bonferroni')

# group contrasts for Figure 4
figure4A <- list(`Fig4A = HL_U - B_U` = c(0,0,0,0,0,0,0,0,0,0,0,0,1/2,1/2,-
1,0))
figure4B <- list(`Fig4B = W_U - HL_U` = c(0,0,0,0,0,0,0,0,0,0,0,0,-1/2,-
1/2,0,1))
figure4_all <- list(`Fig4: HL_U - BW_U` = c(0,0,0,0,0,0,0,0,0,0,0,0,1/2,1/2,-
1/2,-1/2))
#contrast(emmrm1,c(figure4A,figure4B,figure4_all),adjust = "bonferroni")

# group contrasts for Figure 5

figure5A <- list(`Fig5A: HL_N - B_N` = c(0,0,0,0,0,0,0,0,1/2,1/2,-
1,0,0,0,0,0))
figure5B <- list(`Fig5B: W_N - HL_N` = c(0,0,0,0,0,0,0,0,-1/2,-
1/2,0,1,0,0,0,0))
figure5_all <- list(`Fig5: HL_N - BW_N` = c(0,0,0,0,0,0,0,0,1/2,1/2,-1/2,-
1/2,0,0,0,0))
#contrast(emmrm1,c(figure5A,figure5B,figure5_all), adjust = 'bonferroni')

# Figures 4 and 5 combined: all CR targets vs all static targets on static
backgrounds
figure45comb <- list(`Fig45_all: T_BW - T_CR` = c(0,0,0,0,0,0,0,0,1/4,1/4,-
1/4,-1/4,1/4,1/4,-1/4,-1/4))
#contrast(emmrm1,figure45comb)

# group contrasts for Figure 6
B_CR_vs_N <- list(`tgtB: B_HL - BN` = c(0,0,1/2,0,0,0,1/2,0,0,0,-
1,0,0,0,0,0))
W_N_vs_CR <- list(`tgtW: WN - W_HL` = c(0,0,0,-1/2,0,0,0,-
1/2,0,0,0,1,0,0,0,0))
figure6_all <- list(`Fig6: BW_HL - BW_N` = c(0,0,1/4,1/4,0,0,1/4,1/4,0,0,-
1/2,-1/2,0,0,0,0))
figure6A <- list(`Fig6A: BW_H - BW_N` = c(0,0,0,0,0,0,1/2,1/2,0,0,-1/2,-
1/2,0,0,0,0))
figure6B <- list(`Fig6B: BW_L - BW_N` = c(0,0,1/2,1/2,0,0,0,0,0,0,-1/2,-
1/2,0,0,0,0))
#contrast(emmrm1,c(figure6A,figure6B,figure6_all,B_CR_vs_N,W_N_vs_CR), adjust
= 'bonferroni')

# group contrasts for Figure 7
figure7A  <-  list(`Fig7A: SYNC - ASNC` = c (-1/2,1/2,0,0,1/2,-
1/2,0,0,0,0,0,0,0,0,0,0))
figure7B  <-  list(`Fig7B: Tfst - Bfst` = c (1/2,1/2,0,0,-1/2,-
1/2,0,0,0,0,0,0,0,0,0,0))
#contrast(emmrm1,c(figure7A,figure7B),adjust = 'bonferroni')

# all group contrasts from all three figures
```

```
contrast(emmrm1,c(figure3A,figure3B,figure3_all,figure4A,figure4B,figure4_all
,figure5A,figure5B,figure5_all,figure45comb,figure6A,figure6B,figure6_all,B_C
R_vs_N,W_N_vs_CR,figure7A,figure7B),adjust = 'bonferroni')

##  contrast                  estimate      SE   df t.ratio p.value
##  Fig3A: W_UN - B_UN         0.05015 0.00592 5887   8.470  <.0001
##  Fig3B: W_HL - B_HL         0.08282 0.00588 5891  14.080  <.0001
##  Fig3: W_all - B_all        0.06649 0.00420 5874  15.822  <.0001
##  Fig4A = HL_U - B_U         0.01994 0.00733 5861   2.718  0.1119
##  Fig4B = W_U - HL_U         0.02852 0.00714 5889   3.994  0.0011
##  Fig4: HL_U - BW_U         -0.00429 0.00592 5882  -0.725  1.0000
##  Fig5A: HL_N - B_N         -0.07545 0.00737 5834 -10.237  <.0001
##  Fig5B: W_N - HL_N          0.12730 0.00718 5876  17.735  <.0001
##  Fig5: HL_N - BW_N         -0.10138 0.00596 5852 -17.011  <.0001
##  Fig45_all: T_BW - T_CR    -0.05283 0.00421 5880 -12.562  <.0001
##  Fig6A: BW_H - BW_N        -0.04108 0.00584 5858  -7.034  <.0001
##  Fig6B: BW_L - BW_N        -0.00997 0.00588 5834  -1.695  1.0000
##  Fig6: BW_HL - BW_N        -0.02552 0.00508 5842  -5.024  <.0001
##  tgtB: B_HL - BN           -0.04101 0.00734 5850  -5.588  <.0001
##  tgtW: WN - W_HL            0.01004 0.00703 5834   1.428  1.0000
##  Fig7A: SYNC - ASNC         0.07107 0.00621 5834  11.451  <.0001
##  Fig7B: Tfst - Bfst        -0.01008 0.00618 5834  -1.630  1.0000
##
## Degrees-of-freedom method: kenward-roger
## P value adjustment: bonferroni method for 17 tests
```

incidentally, for effectsize, from the model fitted:

```
summary(rm1)

## Linear mixed model fit by maximum likelihood . t-tests use Satterthwaite's
##   method [lmerModLmerTest]
## Formula: rev ~ T * B + (1 | ID/Rn)
##    Data: revDS
##
##       AIC      BIC   logLik deviance df.resid
##   -8688.2  -8561.3   4363.1  -8726.2     5867
##
## Scaled residuals:
##     Min      1Q  Median      3Q     Max
## -4.3771 -0.5760 -0.0826  0.4868 10.6525
##
## Random effects:
##  Groups   Name        Variance  Std.Dev.
##  Rn:ID    (Intercept) 0.0003482 0.01866
##  ID       (Intercept) 0.0107071 0.10347
##  Residual             0.0130049 0.11404
## Number of obs: 5886, groups:  Rn:ID, 70; ID, 10
##
## Fixed effects:
##                Estimate Std. Error         df t value Pr(>|t|)
```

```
## (Intercept)  4.409e-01  3.342e-02  1.073e+01  13.195 5.69e-08 ***
## Tl           8.614e-02  8.749e-03  5.814e+03   9.846  < 2e-16 ***
## Ts           9.260e-02  8.749e-03  5.814e+03  10.584  < 2e-16 ***
## Tw           1.645e-01  8.586e-03  5.851e+03  19.161  < 2e-16 ***
## Bh           8.114e-02  8.749e-03  5.814e+03   9.274  < 2e-16 ***
## Bn           5.649e-02  8.749e-03  5.814e+03   6.457 1.16e-10 ***
## Bu           4.388e-02  8.691e-03  5.834e+03   5.049 4.57e-07 ***
## Tl:Bh       -1.421e-01  1.240e-02  5.814e+03 -11.466  < 2e-16 ***
## Ts:Bh       -1.232e-01  1.212e-02  5.838e+03 -10.160  < 2e-16 ***
## Tw:Bh       -1.013e-01  1.193e-02  5.814e+03  -8.498  < 2e-16 ***
## Tl:Bn       -1.248e-01  1.220e-02  5.814e+03 -10.233  < 2e-16 ***
## Ts:Bn       -3.649e-02  1.220e-02  5.814e+03  -2.992  0.00279 **
## Tw:Bn       -5.656e-02  1.193e-02  5.814e+03  -4.742 2.17e-06 ***
## Tl:Bu       -9.396e-02  1.208e-02  5.820e+03  -7.777 8.75e-15 ***
## Ts:Bu       -1.165e-01  1.216e-02  5.824e+03  -9.579  < 2e-16 ***
## Tw:Bu       -1.399e-01  1.188e-02  5.825e+03 -11.773  < 2e-16 ***
## ---
## Signif. codes:  0 '***' 0.001 '**' 0.01 '*' 0.05 '.' 0.1 ' ' 1

##
## Correlation matrix not shown by default, as p = 16 > 12.
## Use print(x, correlation=TRUE)  or
##     vcov(x)        if you need it
```

```
report_effectsize(rm1)
```

```
## Effect sizes were labelled following Cohen's (1988) recommendations.
##
## small (Std. beta = -0.42, 95% CI [-0.82, -0.03])
## medium (Std. beta = 0.52, 95% CI [0.42, 0.62])
## medium (Std. beta = 0.56, 95% CI [0.46, 0.66])
## large (Std. beta = 0.99, 95% CI [0.89, 1.10])
## small (Std. beta = 0.49, 95% CI [0.39, 0.59])
## small (Std. beta = 0.34, 95% CI [0.24, 0.45])
## small (Std. beta = 0.27, 95% CI [0.16, 0.37])
## large (Std. beta = -0.86, 95% CI [-1.01, -0.71])
## medium (Std. beta = -0.74, 95% CI [-0.89, -0.60])
## medium (Std. beta = -0.61, 95% CI [-0.75, -0.47])
## medium (Std. beta = -0.75, 95% CI [-0.90, -0.61])
## small (Std. beta = -0.22, 95% CI [-0.37, -0.08])
## small (Std. beta = -0.34, 95% CI [-0.48, -0.20])
## medium (Std. beta = -0.57, 95% CI [-0.71, -0.43])
## medium (Std. beta = -0.70, 95% CI [-0.85, -0.56])
## large (Std. beta = -0.85, 95% CI [-0.99, -0.71])
```

We compute below only the custom individual contrasts used in the figures:

```
fig3_ind <- list(`WU - BU` = c(0,0,0,0,0,0,0,0,0,0,0,0,0,0,-1,1),
                    `WN - BN` = c(0,0,0,0,0,0,0,0,0,0,-1,1,0,0,0,0),
                    `WH - BH` = c(0,0,0,0,0,0,-1,1,0,0,0,0,0,0,0,0),
                    `WL - BL` = c(0,0,-1,1,0,0,0,0,0,0,0,0,0,0,0,0))
```

```r
fig4_ind <- list(`LU-BU` = c(0,0,0,0,0,0,0,0,0,0,0,0,0,1,-1,0),
                 `HU-BU` = c(0,0,0,0,0,0,0,0,0,0,0,0,1,0,-1,0),
                 `WU-HU` = c(0,0,0,0,0,0,0,0,0,0,0,0,-1,0,0,1),
                 `WU-LU` = c(0,0,0,0,0,0,0,0,0,0,0,0,0,-1,0,1))
fig5_ind <- list(`LN-BN` = c(0,0,0,0,0,0,0,0,0,1,-1,0,0,0,0,0),
                 `HN-BN` = c(0,0,0,0,0,0,0,0,1,0,-1,0,0,0,0,0),
                 `WN-HN` = c(0,0,0,0,0,0,0,0,-1,0,0,1,0,0,0,0),
                 `WN-LN` = c(0,0,0,0,0,0,0,0,0,-1,0,1,0,0,0,0))
fig6_ind <- list(`BH - BN` = c(0,0,0,0,0,0,1,0,0,0,-1,0,0,0,0,0),
                 `BL - BN` = c(0,0,1,0,0,0,0,0,0,0,-1,0,0,0,0,0),
                 `WN - WH` = c(0,0,0,0,0,0,0,-1,0,0,0,1,0,0,0,0),
                 `WN - WL` = c(0,0,0,-1,0,0,0,0,0,0,0,1,0,0,0,0))
fig7_ind <- list(`LL - HH` = c(0,1,0,0,-1,0,0,0,0,0,0,0,0,0,0,0),
                 `HL - LH` = c(1,0,0,0,0,-1,0,0,0,0,0,0,0,0,0,0),
                 `LL - LH` = c(0,1,0,0,0,-1,0,0,0,0,0,0,0,0,0,0),
                 `LL - HL` = c(-1,1,0,0,0,0,0,0,0,0,0,0,0,0,0,0),
                 `HH - HL` = c(-1,0,0,0,1,0,0,0,0,0,0,0,0,0,0,0),
                 `HH - LH` = c(0,0,0,0,1,-1,0,0,0,0,0,0,0,0,0,0))
contrast(emmrm1,c(fig3_ind,fig4_ind,fig5_ind,fig6_ind,fig7_ind), adjust =
'bonferroni')
```

```
##  contrast  estimate      SE   df t.ratio p.value
##  WU - BU   4.85e-02 0.00834 5868   5.807  <.0001
##  WN - BN   5.18e-02 0.00834 5868   6.214  <.0001
##  WH - BH   9.37e-02 0.00823 5890  11.385  <.0001
##  WL - BL   7.19e-02 0.00834 5868   8.618  <.0001
##  LU-BU     1.60e-02 0.00842 5862   1.902  1.0000
##  HU-BU     2.38e-02 0.00845 5852   2.822  0.1054
##  WU-HU     2.46e-02 0.00828 5880   2.971  0.0656
##  WU-LU     3.24e-02 0.00826 5885   3.928  0.0019
##  LN-BN    -9.48e-02 0.00851 5834 -11.138  <.0001
##  HN-BN    -5.61e-02 0.00851 5834  -6.593  <.0001
##  WN-HN     1.08e-01 0.00834 5868  12.938  <.0001
##  WN-LN     1.47e-01 0.00834 5868  17.573  <.0001
##  BH - BN  -6.20e-02 0.00840 5874  -7.382  <.0001
##  BL - BN  -2.00e-02 0.00851 5834  -2.350  0.4138
##  WN - WH   2.01e-02 0.00811 5834   2.482  0.2883
##  WN - WL  -6.31e-05 0.00811 5834  -0.008  1.0000
##  LL - HH   5.00e-03 0.00851 5834   0.587  1.0000
##  HL - LH  -2.52e-02 0.00897 5834  -2.804  0.1115
##  LL - LH   6.10e-02 0.00876 5834   6.962  <.0001
##  LL - HL   8.61e-02 0.00876 5834   9.833  <.0001
##  HH - HL   8.11e-02 0.00876 5834   9.262  <.0001
##  HH - LH   5.60e-02 0.00876 5834   6.391  <.0001
##
## Degrees-of-freedom method: kenward-roger
## P value adjustment: bonferroni method for 22 tests
```

Table S1: Single participant thresholds per conditions (data in Figures 3 to 7)

	ID	T	B	CL	VAR	N	MEAN	CI	SD	SE
1	1	H	L	7	rev	36	0.430	0.012	0.035	0.006
2	1	H	H	1	rev	36	0.467	0.019	0.057	0.009
3	1	H	N	2	rev	36	0.429	0.013	0.038	0.006
4	1	H	U	3	rev	36	0.366	0.011	0.034	0.006
5	1	L	L	4	rev	36	0.488	0.020	0.059	0.010
6	1	L	H	8	rev	36	0.415	0.020	0.058	0.010
7	1	L	N	5	rev	36	0.383	0.007	0.021	0.004
8	1	L	U	6	rev	36	0.429	0.018	0.055	0.009
9	1	B	L	10	rev	36	0.472	0.016	0.048	0.008
10	1	B	H	9	rev	36	0.512	0.015	0.045	0.007
11	1	B	N	11	rev	36	0.455	0.013	0.038	0.006
12	1	B	U	12	rev	36	0.430	0.012	0.035	0.006
13	1	W	L	14	rev	72	0.573	0.017	0.071	0.008
14	1	W	H	13	rev	72	0.675	0.012	0.049	0.006
15	1	W	N	15	rev	72	0.501	0.017	0.072	0.008
16	1	W	U	16	rev	72	0.442	0.012	0.052	0.006
17	2	H	L	7	rev	36	0.505	0.026	0.076	0.013
18	2	H	H	1	rev	36	0.471	0.040	0.118	0.020
19	2	H	N	2	rev	36	0.552	0.041	0.120	0.020
20	2	H	U	3	rev	36	0.556	0.040	0.119	0.020
21	2	L	L	4	rev	36	0.501	0.023	0.068	0.011
22	2	L	H	8	rev	36	0.598	0.058	0.171	0.028
23	2	L	N	5	rev	36	0.420	0.024	0.072	0.012
24	2	L	U	6	rev	36	0.454	0.039	0.117	0.019
25	2	B	L	10	rev	36	0.425	0.038	0.113	0.019
26	2	B	H	9	rev	36	0.317	0.026	0.077	0.013
27	2	B	N	11	rev	36	0.501	0.033	0.097	0.016
28	2	B	U	12	rev	36	0.440	0.016	0.048	0.008
29	2	W	L	14	rev	36	0.492	0.048	0.141	0.023
30	2	W	H	13	rev	36	0.501	0.056	0.166	0.028
31	2	W	N	15	rev	36	0.591	0.036	0.107	0.018
32	2	W	U	16	rev	36	0.637	0.044	0.130	0.022
33	3	H	L	7	rev	36	0.407	0.019	0.057	0.010
34	3	H	H	1	rev	36	0.488	0.025	0.073	0.012
35	3	H	N	2	rev	36	0.442	0.014	0.042	0.007
36	3	H	U	3	rev	36	0.419	0.025	0.073	0.012
37	3	L	L	4	rev	36	0.430	0.026	0.077	0.013
38	3	L	H	8	rev	36	0.393	0.021	0.063	0.010
39	3	L	N	5	rev	36	0.461	0.018	0.053	0.009

40	3	L	U	6	rev	36	0.484	0.037	0.108	0.018
41	3	B	L	10	rev	36	0.511	0.029	0.086	0.014
42	3	B	H	9	rev	36	0.408	0.015	0.045	0.008
43	3	B	N	11	rev	36	0.443	0.028	0.082	0.014
44	3	B	U	12	rev	36	0.399	0.013	0.039	0.006
45	3	W	L	14	rev	36	0.679	0.045	0.133	0.022
46	3	W	H	13	rev	36	0.660	0.059	0.174	0.029
47	3	W	N	15	rev	36	0.672	0.043	0.128	0.021
48	3	W	U	16	rev	36	0.487	0.018	0.053	0.009
49	4	H	L	7	rev	36	0.481	0.035	0.104	0.017
50	4	H	H	1	rev	36	0.688	0.034	0.099	0.017
51	4	H	N	2	rev	36	0.599	0.024	0.071	0.012
52	4	H	U	3	rev	36	0.598	0.037	0.111	0.018
53	4	L	L	4	rev	36	0.617	0.038	0.112	0.019
54	4	L	H	8	rev	36	0.498	0.023	0.067	0.011
55	4	L	N	5	rev	36	0.537	0.034	0.101	0.017
56	4	L	U	6	rev	36	0.545	0.021	0.061	0.010
57	4	B	L	10	rev	36	0.640	0.035	0.103	0.017
58	4	B	H	9	rev	36	0.622	0.040	0.118	0.020
59	4	B	N	11	rev	36	0.582	0.035	0.103	0.017
60	4	B	U	12	rev	36	0.591	0.027	0.078	0.013
61	4	W	L	14	rev	36	0.719	0.053	0.156	0.026
62	4	W	H	13	rev	36	0.631	0.048	0.143	0.024
63	4	W	N	15	rev	36	0.858	0.056	0.167	0.028
64	4	W	U	16	rev	36	0.640	0.038	0.112	0.019
65	5	H	L	7	rev	36	0.178	0.027	0.079	0.013
66	5	H	H	1	rev	36	0.313	0.075	0.221	0.037
67	5	H	N	2	rev	36	0.228	0.028	0.083	0.014
68	5	H	U	3	rev	48	0.312	0.021	0.072	0.010
69	5	L	L	4	rev	36	0.215	0.020	0.060	0.010
70	5	L	H	8	rev	36	0.182	0.029	0.085	0.014
71	5	L	N	5	rev	36	0.189	0.021	0.061	0.010
72	5	L	U	6	rev	54	0.278	0.017	0.061	0.008
73	5	B	L	10	rev	36	0.290	0.031	0.092	0.015
74	5	B	H	9	rev	60	0.353	0.026	0.102	0.013
75	5	B	N	11	rev	36	0.302	0.021	0.061	0.010
76	5	B	U	12	rev	36	0.235	0.030	0.088	0.015
77	5	W	L	14	rev	36	0.331	0.044	0.129	0.022
78	5	W	H	13	rev	36	0.232	0.029	0.085	0.014
79	5	W	N	15	rev	36	0.271	0.041	0.121	0.020
80	5	W	U	16	rev	36	0.294	0.041	0.122	0.020
81	6	H	L	7	rev	36	0.533	0.033	0.097	0.016

82	6	H	H	1	rev	36	0.577	0.038	0.113	0.019
83	6	H	N	2	rev	36	0.693	0.023	0.069	0.012
84	6	H	U	3	rev	36	0.536	0.034	0.101	0.017
85	6	L	L	4	rev	36	0.558	0.028	0.083	0.014
86	6	L	H	8	rev	36	0.638	0.051	0.150	0.025
87	6	L	N	5	rev	36	0.595	0.043	0.126	0.021
88	6	L	U	6	rev	36	0.549	0.023	0.067	0.011
89	6	B	L	10	rev	36	0.812	0.060	0.177	0.029
90	6	B	H	9	rev	36	0.610	0.030	0.088	0.015
91	6	B	N	11	rev	36	0.745	0.058	0.173	0.029
92	6	B	U	12	rev	36	0.502	0.042	0.125	0.021
93	6	W	L	14	rev	36	0.909	0.069	0.203	0.034
94	6	W	H	13	rev	36	0.599	0.041	0.120	0.020
95	6	W	N	15	rev	36	0.540	0.027	0.080	0.013
96	6	W	U	16	rev	36	0.544	0.022	0.064	0.011
97	8	H	H	1	rev	36	0.574	0.037	0.109	0.018
98	8	H	N	2	rev	36	0.490	0.025	0.073	0.012
99	8	H	U	3	rev	36	0.477	0.033	0.096	0.016
100	8	L	L	4	rev	36	0.557	0.028	0.082	0.014
101	8	L	N	5	rev	36	0.437	0.016	0.046	0.008
102	8	L	U	6	rev	36	0.505	0.024	0.071	0.012
103	8	B	L	10	rev	36	0.492	0.020	0.059	0.010
104	8	B	H	9	rev	36	0.510	0.029	0.084	0.014
105	8	B	N	11	rev	36	0.519	0.021	0.063	0.010
106	8	B	U	12	rev	36	0.508	0.024	0.071	0.012
107	8	W	L	14	rev	36	0.558	0.020	0.060	0.010
108	8	W	H	13	rev	36	0.537	0.025	0.075	0.013
109	8	W	N	15	rev	36	0.753	0.044	0.131	0.022
110	8	W	U	16	rev	36	0.498	0.023	0.067	0.011
111	9	H	L	7	rev	36	0.514	0.034	0.099	0.017
112	9	H	H	1	rev	36	0.596	0.046	0.137	0.023
113	9	H	N	2	rev	36	0.587	0.028	0.082	0.014
114	9	H	U	3	rev	36	0.569	0.026	0.077	0.013
115	9	L	L	4	rev	36	0.869	0.046	0.135	0.023
116	9	L	H	8	rev	36	0.537	0.045	0.133	0.022
117	9	L	N	5	rev	36	0.643	0.042	0.124	0.021
118	9	L	U	6	rev	36	0.675	0.034	0.099	0.017
119	9	B	L	10	rev	36	0.664	0.059	0.176	0.029
120	9	B	H	9	rev	36	0.420	0.027	0.081	0.013
121	9	B	N	11	rev	36	0.987	0.061	0.180	0.030
122	9	B	U	12	rev	36	0.622	0.030	0.088	0.015
123	9	W	L	14	rev	36	0.713	0.036	0.105	0.018

124	9	W	H	13	rev	36	0.762	0.036	0.107	0.018
125	9	W	N	15	rev	36	0.763	0.025	0.075	0.012
126	9	W	U	16	rev	36	0.615	0.021	0.062	0.010
127	10	H	L	7	rev	36	0.500	0.017	0.050	0.008
128	10	H	H	1	rev	36	0.533	0.033	0.097	0.016
129	10	H	N	2	rev	36	0.507	0.017	0.051	0.009
130	10	H	U	3	rev	36	0.540	0.019	0.057	0.010
131	10	L	L	4	rev	36	0.549	0.017	0.049	0.008
132	10	L	H	8	rev	36	0.510	0.026	0.076	0.013
133	10	L	N	5	rev	36	0.525	0.024	0.072	0.012
134	10	L	U	6	rev	36	0.442	0.023	0.067	0.011
135	10	B	L	10	rev	36	0.576	0.028	0.081	0.014
136	10	B	H	9	rev	36	0.598	0.021	0.061	0.010
137	10	B	N	11	rev	36	0.522	0.024	0.071	0.012
138	10	B	U	12	rev	36	0.463	0.019	0.056	0.009
139	10	W	L	14	rev	36	0.588	0.020	0.060	0.010
140	10	W	H	13	rev	36	0.605	0.021	0.063	0.010
141	10	W	N	15	rev	36	0.678	0.034	0.102	0.017
142	10	W	U	16	rev	36	0.515	0.016	0.048	0.008
143	11	H	L	7	rev	36	0.394	0.018	0.052	0.009
144	11	H	H	1	rev	36	0.492	0.017	0.051	0.008
145	11	H	N	2	rev	36	0.428	0.017	0.050	0.008
146	11	H	U	3	rev	36	0.438	0.014	0.040	0.007
147	11	L	L	4	rev	36	0.467	0.028	0.083	0.014
148	11	L	H	8	rev	36	0.396	0.020	0.059	0.010
149	11	L	N	5	rev	36	0.378	0.014	0.043	0.007
150	11	L	U	6	rev	36	0.376	0.013	0.039	0.006
151	11	B	L	10	rev	36	0.434	0.018	0.053	0.009
152	11	B	H	9	rev	36	0.485	0.026	0.076	0.013
153	11	B	N	11	rev	36	0.460	0.028	0.084	0.014
154	11	B	U	12	rev	36	0.401	0.031	0.091	0.015
155	11	W	L	14	rev	36	0.483	0.025	0.073	0.012
156	11	W	H	13	rev	36	0.518	0.031	0.091	0.015
157	11	W	N	15	rev	36	0.490	0.026	0.076	0.013
158	11	W	U	16	rev	36	0.446	0.028	0.082	0.014

Table S2: Summary statistics per combination of T and B factor levels (condition)

T fctr	B fctr	Cd #	var	n	Mean	ci	sd	se
H	L	7	rev	324	0.438	0.014	0.128	0.007
H	H	1	rev	360	0.520	0.015	0.149	0.008
H	N	2	rev	360	0.495	0.014	0.140	0.007
H	U	3	rev	372	0.476	0.013	0.124	0.006
L	L	4	rev	360	0.525	0.018	0.176	0.009
L	H	8	rev	324	0.463	0.018	0.165	0.009
L	N	5	rev	360	0.457	0.015	0.146	0.008
L	U	6	rev	378	0.464	0.013	0.132	0.007
B	L	10	rev	360	0.532	0.018	0.176	0.009
B	H	9	rev	384	0.475	0.013	0.132	0.007
B	N	11	rev	360	0.552	0.022	0.208	0.011
B	U	12	rev	360	0.459	0.013	0.128	0.007
W	L	14	rev	396	0.602	0.018	0.186	0.009
W	H	13	rev	396	0.581	0.017	0.172	0.009
W	N	15	rev	396	0.602	0.019	0.190	0.010
W	U	16	rev	396	0.506	0.013	0.127	0.006

1-16 of 16 rows

Table S3: Parameters of the model. Formula: rev ~ T*B + (1|ID/Rn)

Fixed Effects

Parameter	Coefficient	SE	95% CI	t(5867)	p
(Intercept)	0.44	0.03	[0.38, 0.51]	13.19	< .001
T [L]	0.09	8.75e-03	[0.07, 0.10]	9.85	< .001
T [B]	0.09	8.75e-03	[0.08, 0.11]	10.58	< .001
T [W]	0.16	8.59e-03	[0.15, 0.18]	19.16	< .001
B [H]	0.08	8.75e-03	[0.06, 0.10]	9.27	< .001
B [N]	0.06	8.75e-03	[0.04, 0.07]	6.46	< .001
B [U]	0.04	8.69e-03	[0.03, 0.06]	5.05	< .001
T [L] × B [H]	-0.14	0.01	[-0.17, -0.12]	-11.47	< .001
T [B] × B [H]	-0.12	0.01	[-0.15, -0.10]	-10.16	< .001
T [W] × B [H]	-0.10	0.01	[-0.12, -0.08]	-8.50	< .001
T [L] × B [N]	-0.12	0.01	[-0.15, -0.10]	-10.23	< .001
T [B] × B [N]	-0.04	0.01	[-0.06, -0.01]	-2.99	0.003
T [W] × B [N]	-0.06	0.01	[-0.08, -0.03]	-4.74	< .001
T [L] × B [U]	-0.09	0.01	[-0.12, -0.07]	-7.78	< .001
T [B] × B [U]	-0.12	0.01	[-0.14, -0.09]	-9.58	< .001
T [W] × B [U]	-0.14	0.01	[-0.16, -0.12]	-11.77	< .001

Random Effects

Parameter	Coefficient
SD (Intercept: Rn:ID)	0.02
SD (Intercept: ID)	0.10
SD (Residual)	0.11

Uncertainty intervals (equal-tailed) and p-values (two-tailed) computed using a Wald t-distribution approximation.

Random Effects

Parameter	Coefficient
SD (Intercept: Rn:ID)	0.02
SD (Intercept: ID)	0.10
SD (Residual)	0.11

Uncertainty intervals (equal-tailed) and p-values (two-tailed) computed using a Wald t-distribution approximation.

Table S4: Pairwise contrasts corrected for multiple comparison

Marginal Contrasts Analysis

Level1	Level2	Difference	95% CI	SE	df	t	p
H L	H H	-0.08	[-0.11, -0.05]	8.76e-03	5833.82	-9.26	< .001
H L	H N	-0.06	[-0.09, -0.03]	8.76e-03	5833.82	-6.45	< .001
H L	H U	-0.04	[-0.07, -0.01]	8.70e-03	5852.15	-5.04	< .001
H L	L L	-0.09	[-0.12, -0.06]	8.76e-03	5833.82	-9.83	< .001
H L	L H	-0.03	[-0.06, 0.01]	8.97e-03	5833.66	-2.80	0.608
H L	L N	-0.02	[-0.05, 0.01]	8.76e-03	5833.82	-2.03	> .999
H L	L U	-0.04	[-0.07, -0.01]	8.68e-03	5862.11	-4.16	0.004
H L	B L	-0.09	[-0.12, -0.06]	8.76e-03	5833.82	-10.57	< .001
H L	B H	-0.05	[-0.08, -0.02]	8.65e-03	5873.80	-5.85	< .001
H L	B N	-0.11	[-0.14, -0.08]	8.76e-03	5833.82	-12.85	< .001
H L	B U	-0.02	[-0.05, 0.01]	8.76e-03	5833.82	-2.29	> .999
H L	W L	-0.16	[-0.19, -0.13]	8.60e-03	5868.12	-19.13	< .001
H L	W H	-0.14	[-0.17, -0.11]	8.60e-03	5868.12	-16.79	< .001
H L	W N	-0.16	[-0.19, -0.13]	8.60e-03	5868.12	-19.13	< .001
H L	W U	-0.07	[-0.10, -0.04]	8.60e-03	5868.12	-7.97	< .001
H H	H N	0.02	[-0.01, 0.05]	8.51e-03	5833.66	2.90	0.454
H H	H U	0.04	[0.01, 0.07]	8.45e-03	5852.17	4.41	0.001
H H	L H	0.06	[0.03, 0.09]	8.76e-03	5833.82	6.39	< .001
H H	L N	0.06	[0.03, 0.09]	8.51e-03	5833.66	7.44	< .001
H H	L U	0.05	[0.02, 0.07]	8.42e-03	5862.37	5.35	< .001
H H	B H	0.03	[0.00, 0.06]	8.40e-03	5874.42	3.64	0.033
H H	B N	-0.03	[-0.06, 0.00]	8.51e-03	5833.66	-3.70	0.027
H H	B U	0.06	[0.03, 0.09]	8.51e-03	5833.66	7.18	< .001
H H	W H	-0.06	[-0.09, -0.03]	8.34e-03	5867.94	-7.57	< .001
H H	W N	-0.08	[-0.11, -0.05]	8.34e-03	5867.94	-9.98	< .001
H H	W U	0.01	[-0.02, 0.04]	8.34e-03	5867.94	1.52	> .999
H N	H U	0.01	[-0.02, 0.04]	8.45e-03	5852.17	1.49	> .999
H N	L N	0.04	[0.01, 0.07]	8.51e-03	5833.66	4.54	< .001
H N	L U	0.02	[-0.01, 0.05]	8.42e-03	5862.37	2.42	> .999
H N	B N	-0.06	[-0.09, -0.03]	8.51e-03	5833.66	-6.59	< .001
H N	B U	0.04	[0.01, 0.07]	8.51e-03	5833.66	4.28	0.002
H N	W N	-0.11	[-0.14, -0.08]	8.34e-03	5867.94	-12.94	< .001
H N	W U	-0.01	[-0.04, 0.02]	8.34e-03	5867.94	-1.44	> .999
H U	L U	7.82e-03	[-0.02, 0.04]	8.34e-03	5845.74	0.94	> .999
H U	B U	0.02	[-0.01, 0.05]	8.45e-03	5852.17	2.82	0.575
H U	W U	-0.02	[-0.05, 0.00]	8.28e-03	5879.51	-2.97	0.358
L L	H H	5.00e-03	[-0.03, 0.04]	8.51e-03	5833.66	0.59	> .999
L L	H N	0.03	[0.00, 0.06]	8.51e-03	5833.66	3.48	0.060
L L	H U	0.04	[0.01, 0.07]	8.45e-03	5852.17	5.00	< .001
L L	L H	0.06	[0.03, 0.09]	8.76e-03	5833.82	6.96	< .001
L L	L N	0.07	[0.04, 0.10]	8.51e-03	5833.66	8.03	< .001
L L	L U	0.05	[0.02, 0.08]	8.42e-03	5862.37	5.94	< .001
L L	B L	-6.46e-03	[-0.04, 0.02]	8.51e-03	5833.66	-0.76	> .999
L L	B H	0.04	[0.01, 0.07]	8.40e-03	5874.42	4.23	0.003
L L	B N	-0.03	[-0.06, 0.00]	8.51e-03	5833.66	-3.11	0.227
L L	B U	0.07	[0.04, 0.10]	8.51e-03	5833.66	7.77	< .001
L L	W L	-0.08	[-0.11, -0.05]	8.34e-03	5867.94	-9.39	< .001
L L	W H	-0.06	[-0.09, -0.03]	8.34e-03	5867.94	-6.97	< .001
L L	W N	-0.08	[-0.11, -0.05]	8.34e-03	5867.94	-9.38	< .001
L L	W U	0.02	[-0.01, 0.05]	8.34e-03	5867.94	2.12	> .999
L H	H N	-0.03	[-0.06, 0.00]	8.76e-03	5833.82	-3.58	0.042
L H	H U	-0.02	[-0.05, 0.01]	8.70e-03	5852.15	-2.15	> .999
L H	L N	7.34e-03	[-0.02, 0.04]	8.76e-03	5833.82	0.84	> .999
L H	L U	-0.01	[-0.04, 0.02]	8.68e-03	5862.11	-1.26	> .999
L H	B H	-0.03	[-0.06, 0.01]	8.65e-03	5873.80	-2.94	0.397
L H	B N	-0.09	[-0.12, -0.06]	8.76e-03	5833.82	-9.98	< .001
L H	B U	5.12e-03	[-0.03, 0.04]	8.76e-03	5833.82	0.58	> .999
L H	W H	-0.12	[-0.15, -0.09]	8.60e-03	5868.12	-13.86	< .001
L H	W N	-0.14	[-0.17, -0.11]	8.60e-03	5868.12	-16.20	< .001
L H	W U	-0.04	[-0.07, -0.01]	8.60e-03	5868.12	-5.04	< .001
L N	H U	-0.03	[-0.06, 0.00]	8.45e-03	5852.17	-3.08	0.246
L N	L U	-0.02	[-0.05, 0.01]	8.42e-03	5862.37	-2.17	> .999
L N	B N	-0.09	[-0.12, -0.06]	8.51e-03	5833.66	-11.14	< .001
L N	B U	-2.22e-03	[-0.03, 0.03]	8.51e-03	5833.66	-0.26	> .999
L N	W N	-0.15	[-0.18, -0.12]	8.34e-03	5867.94	-17.57	< .001
L N	W U	-0.05	[-0.08, -0.02]	8.34e-03	5867.94	-6.07	< .001
L U	B U	0.02	[-0.01, 0.05]	8.42e-03	5862.37	1.90	> .999
L U	W U	-0.03	[-0.06, 0.00]	8.26e-03	5884.98	-3.93	0.010
B L	H H	0.01	[-0.02, 0.04]	8.51e-03	5833.66	1.35	> .999
B L	H N	0.04	[0.01, 0.07]	8.51e-03	5833.66	4.24	0.003

B L	H U	0.05	[0.02, 0.08]	8.45e-03	5852.17	5.76	< .001
B L	L H	0.07	[0.04, 0.10]	8.76e-03	5833.82	7.70	< .001
B L	L N	0.07	[0.04, 0.10]	8.51e-03	5833.66	8.79	< .001
B L	L U	0.06	[0.03, 0.09]	8.42e-03	5862.37	6.71	< .001
B L	B H	0.04	[0.01, 0.07]	8.40e-03	5874.42	5.00	< .001
B L	B N	-0.02	[-0.05, 0.01]	8.51e-03	5833.66	-2.35	> .999
B L	B U	0.07	[0.04, 0.10]	8.51e-03	5833.66	8.53	< .001
B L	W L	-0.07	[-0.10, -0.04]	8.34e-03	5867.94	-8.62	< .001
B L	W H	-0.05	[-0.08, -0.02]	8.34e-03	5867.94	-6.20	< .001
B L	W N	-0.07	[-0.10, -0.04]	8.34e-03	5867.94	-8.61	< .001
B L	W U	0.02	[-0.01, 0.05]	8.34e-03	5867.94	2.89	0.465
B H	H N	-5.91e-03	[-0.04, 0.02]	8.40e-03	5874.42	-0.70	> .999
B H	H U	6.70e-03	[-0.02, 0.04]	8.32e-03	5860.29	0.81	> .999
B H	L N	0.03	[0.00, 0.06]	8.40e-03	5874.42	3.90	0.012
B H	L U	0.01	[-0.01, 0.04]	8.28e-03	5849.24	1.75	> .999
B H	B N	-0.06	[-0.09, -0.03]	8.40e-03	5874.42	-7.38	< .001
B H	B U	0.03	[0.00, 0.06]	8.40e-03	5874.42	3.64	0.033
B H	W H	-0.09	[-0.12, -0.06]	8.23e-03	5889.94	-11.38	< .001
B H	W N	-0.11	[-0.14, -0.08]	8.23e-03	5889.94	-13.83	< .001
B H	W U	-0.02	[-0.05, 0.01]	8.23e-03	5889.94	-2.18	> .999
B N	H U	0.07	[0.04, 0.10]	8.45e-03	5852.17	8.13	< .001
B N	L U	0.08	[0.05, 0.11]	8.42e-03	5862.37	9.09	< .001
B N	B U	0.09	[0.06, 0.12]	8.51e-03	5833.66	10.88	< .001
B N	W N	-0.05	[-0.08, -0.02]	8.34e-03	5867.94	-6.21	< .001
B N	W U	0.04	[0.01, 0.07]	8.34e-03	5867.94	5.29	< .001
B U	W U	-0.05	[-0.08, -0.02]	8.34e-03	5867.94	-5.81	< .001
W L	H H	0.08	[0.05, 0.11]	8.34e-03	5867.94	9.99	< .001
W L	H N	0.11	[0.08, 0.14]	8.34e-03	5867.94	12.95	< .001
W L	H U	0.12	[0.09, 0.15]	8.28e-03	5879.51	14.56	< .001
W L	L H	0.14	[0.11, 0.17]	8.60e-03	5868.12	16.21	< .001
W L	L N	0.15	[0.12, 0.18]	8.34e-03	5867.94	17.58	< .001
W L	L U	0.13	[0.10, 0.16]	8.26e-03	5884.98	15.56	< .001
W L	B H	0.11	[0.08, 0.14]	8.23e-03	5889.94	13.84	< .001
W L	B N	0.05	[0.02, 0.08]	8.34e-03	5867.94	6.22	< .001
W L	B U	0.14	[0.12, 0.17]	8.34e-03	5867.94	17.31	< .001
W L	W H	0.02	[-0.01, 0.05]	8.11e-03	5833.66	2.49	> .999
W L	W N	6.31e-05	[-0.03, 0.03]	8.11e-03	5833.66	7.78e-03	> .999
W L	W U	0.10	[0.07, 0.12]	8.11e-03	5833.66	11.83	< .001
W H	H N	0.09	[0.06, 0.12]	8.34e-03	5867.94	10.52	< .001
W H	H U	0.10	[0.07, 0.13]	8.28e-03	5879.51	12.12	< .001
W H	L N	0.13	[0.10, 0.16]	8.34e-03	5867.94	15.16	< .001
W H	L U	0.11	[0.08, 0.14]	8.26e-03	5884.98	13.11	< .001
W H	B N	0.03	[0.00, 0.06]	8.34e-03	5867.94	3.80	0.018
W H	B U	0.12	[0.09, 0.15]	8.34e-03	5867.94	14.89	< .001
W H	W N	-0.02	[-0.05, 0.01]	8.11e-03	5833.66	-2.48	> .999
W H	W U	0.08	[0.05, 0.10]	8.11e-03	5833.66	9.34	< .001
W N	H U	0.12	[0.09, 0.15]	8.28e-03	5879.51	14.55	< .001
W N	L U	0.13	[0.10, 0.16]	8.26e-03	5884.98	15.55	< .001
W N	B U	0.14	[0.11, 0.17]	8.34e-03	5867.94	17.31	< .001
W N	W U	0.10	[0.07, 0.12]	8.11e-03	5833.66	11.83	< .001

Marginal contrasts estimated at T, B
p-value adjustment method: Bonferroni
